# Visualising redox-driven metal–oxide interface dynamics in Ni/GDC electrodes *via in situ* Auger–Meitner electron microscopy

**DOI:** 10.1039/d6ta04877c

**Published:** 2026-10-07

**Authors:** Christian Melcher, Andreas Nenning, Stanislaus Breitwieser, Kirsten Rath, Luca Balice, Jonathan Rollenitz, Martin Bram, Christoph Rameshan, Alexander Karl Opitz

**Affiliations:** a TU Wien, Institute of Chemical Technologies and Analytics Austria christian.melcher@tuwien.ac.at alexander.opitz@tuwien.ac.at; b Forschungszentrum Jülich, Institute of Energy Materials and Devices Germany; c Montanuniversität Leoben, Chair of Physical Chemistry Austria

## Abstract

Metal/oxide interfaces play a decisive role in both high-temperature electrochemical energy conversion using solid oxide cells (SOCs) and heterogeneous catalysis. However, their morphological stability under dynamic redox conditions remains poorly understood. In the present work, we investigate model electrodes of Ni/GDC (GDC = Gd-doped ceria = Gd_0.1_Ce_0.9_O_2−*δ*_) composites in thin-film and porous form. Electrochemical polarization of these electrodes enables electrochemical control of the oxygen chemical potential in ceria and thus provides direct experimental access to redox-driven interface dynamics independent of the gas-phase composition. While morphological degradation in Ni/GDC is often discussed in the context of electrochemical polarization, the underlying driving forces are more general and arise from changes in local oxygen chemical potential, metal oxidation state, and oxide defect chemistry. In this study, we present a multi-method fundamental analysis that enables well-defined control of the oxygen chemical potential in ceria while simultaneously resolving surface chemistry and morphology of Ni/GDC. By combining the in-house developed Electrochemical oXygen Activity ConTrol (EXACT) method with *in situ* Auger–Meitner electron microscopy (AMEM), we directly visualize redox-driven interface dynamics over an exceptionally wide effective *p*O_2_ range. The results on the studied Ni/ceria system clearly show that upon consecutive oxidation and reduction of Ni and NiO (redox-cycling), dewetting – one of the main degradation mechanisms in solid oxide cells – is strongly accelerated. Notably, formation of only a thin NiO interface layer is sufficient to trigger this destabilization, without requiring full oxidation of Ni. However, while conditions are kept more reducing than the Ni/NiO threshold, Ni remains morphologically stable, even though the effective *p*O_2_ varies by more than 10 orders of magnitude. These trends are observed for both thin-film model systems and 3D porous Ni/GDC composites. Using near ambient pressure X-ray photoelectron spectroscopy (NAP-XPS) on thin-film samples, we demonstrate that our results still hold true when the electrodes are exposed to gas atmospheres such as H_2_/H_2_O and CO/CO_2_. In addition to Ni redistribution, pronounced ceria mobility is observed, including migration of a Ce-oxide species onto Ni under strongly reducing conditions, and Ce enrichment near Ni/NiO triple-phase boundaries under oxidising conditions, thus highlighting the active role of the oxide support in redox-driven morphology evolution.

## Abstract 2

*In situ* Auger–Meitner electron microscopy reveals voltage-driven morphological evolution in solid oxide cell electrodes.

## Graphical abstract

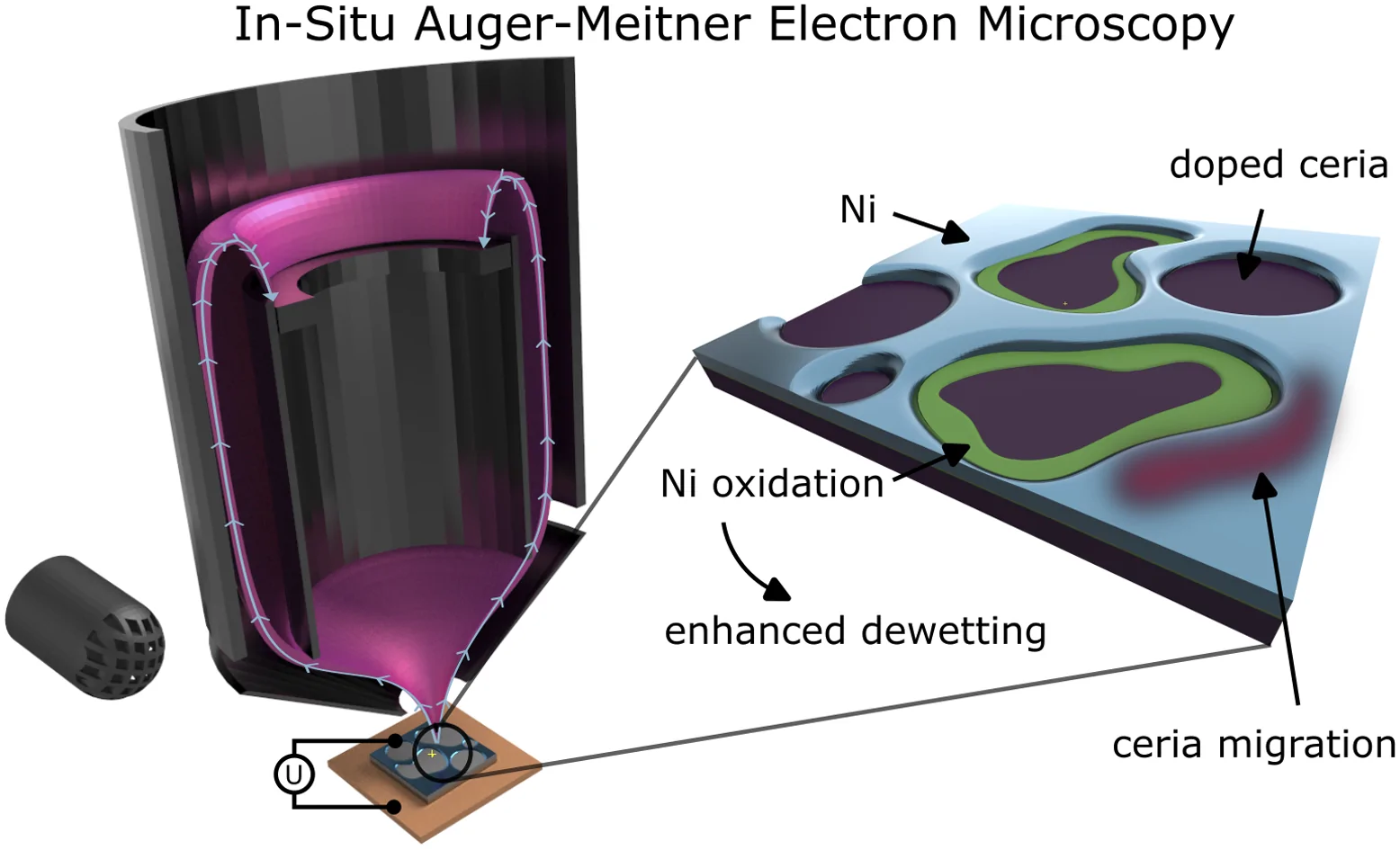

## 1 Introduction

With global energy demand and CO_2_ levels rising, conversion and storage of renewable energy remain key challenges in our society. Solid oxide cells (SOCs) are a promising and highly energy-efficient technology for this task, particularly in applications such as hydrogen production and CO_2_ electrolysis. At the same time, the decarbonization of the chemical industry critically depends on the development of efficient and stable heterogeneous catalysts, as they are indispensable not only for energy-related processes but also for the sustainable production of resources required for everyday materials and products. Notably, typical metal/ceramic-based SOC electrode materials are closely related to metal/oxide catalyst systems used in heterogeneous catalysis. In both application scenarios, the morphological change of these functional composite materials is a central problem that can lead to a loss of the desired properties. Therefore, a fundamental understanding of the mobility of the components in both SOC composite electrodes and oxide-supported metal catalysts is of immense importance.

Composite electrodes are at the core of solid oxide cell (SOC) technology, enabling high electrochemical performance through the intimate coupling of an electronic with an ionic conductor, at least one of them also exhibiting a catalytically active phase. Ni-based composites such as Ni/YSZ (Y-stabilized ZrO_2_) and Ni/GDC (Gd-doped CeO_2_) remain benchmark fuel electrodes for both SOFC and SOEC operation.^1–6^ However, their long-term performance is limited by intrinsic instability of the composite microstructure under operating conditions.^7–9^ For Ni/YSZ composites, electrochemical polarization induces pronounced morphology changes including Ni migration,^10^ coarsening,^11^ agglomeration,^12^ loss of percolation^13,14^ and changes in wettability on YSZ,^15^ especially in electrolysis operation. These degradation mechanisms represent a major bottleneck for durable SOC operation and continue to challenge the large-scale implementation of this otherwise highly promising technology.

Beyond conventional Ni/YSZ composites, alternative ceramic phases have been explored to further enhance the electrochemical activity of SOC fuel electrodes. Gd-doped CeO_2_ (GDC) represents an important improvement, as it introduces additional functionality within the ceramic phase.^16,17^ Under reducing conditions and, crucially, under cathodic polarization, the oxygen chemical potential (*µ*_O_) within GDC is decreased, driving partial reduction of Ce^4+^ to Ce^3+^ and thus turning GDC into a mixed ionic electronic conductor.^7,17–19^ This polarization-induced change in defect chemistry affects the material's transport properties and, in contrast to purely ionic YSZ, extends the electrochemically active region way beyond the triple phase boundary. Moreover, these defect chemical changes affect interfacial energies, thereby influencing the morphological stability of the composite electrode under operation. Despite the technological relevance of Ni/GDC systems, fundamental insights into how controlled *µ*_O_ variations during operation influence morphology and interface evolution remain scarce with only some exceptions.^8,20,21^ Overall, the morphological changes of Ni/GDC are more complex than for Ni/YSZ. Especially in electrolysis operation, electrochemical polarisation leads to chemical expansion of GDC, which induces strain. Moreover, GDC overgrow on Ni was reported under cathodic polarization in high humidity conditions.^20^ Furthermore, the origin of Ni migration is not clearly resolved as both, gas phase transport and surface diffusion are hypothesized. It is therefore imperative to understand the fundamental relationship between voltage-induced variations in *µ*_O_ and the associated morphological changes.

In addition to SOC applications, catalyst particles supported on ceria-based oxides represent also a key material class in heterogeneous catalysis, for example in CO oxidation,^22–26^ water–gas shift,^27–29^ dry reforming of methane,^30^ and methanol synthesis,^31^ among others. Under such reaction conditions, the catalyst is exposed to strongly non-equilibrium gas atmospheres, resulting in locally strongly varying effective *p*O_2_ (with *µ*_O_-gradients similar to electrochemically polarized electrodes), which can even result in *µ*_O_-oscillations at the catalyst surface.^32–35^ The related dynamic Ce^4+^/Ce^3+^ redox transition^7,17–19^ and the associated changes in point defect concentrations of ceria strongly affect metal dispersion, sintering behaviour, metal–support-interaction strength and, ultimately, catalytic activity and stability. Consequently, morphological stability issues observed in Ni/GDC electrodes are closely connected to degradation phenomena in ceria-supported metal catalysts.

Beyond these parallels in degradation phenomena, the effect of non-faradaic electrochemical modification of catalytic activity (NEMCA) – often also called electrochemical promotion of catalysis (EPOC) – establishes a direct conceptual bridge between polarized SOC cermet electrodes and heterogeneous catalysis, as it enables the electrochemical control of catalytic activity *via* changes in the chemical potential of oxygen and the metal–support-interaction.^36–38^ In this context, Ni/GDC represents a prototypical model system at the interface between electrochemistry and catalysis, ideally suited for studying how electrochemical polarization dynamically modulates interfacial chemistry, catalytic activity and morphological stability. In addition, these processes are also highly relevant for defect-chemical and atomistic modeling of metal/ceria systems.

To access these coupled electrochemical, catalytic, and morphological phenomena under well-defined conditions, advanced *in situ* surface sensitive analysis techniques are required that allow for simultaneous control of *µ*_O_ – and therefore the effective *p*O_2_ – at elevated temperatures. Although advanced *in situ* techniques such as *in situ* TEM, environmental SEM, and near ambient pressure (NAP) XPS are available, each comes with inherent limitations. *In situ* TEM offers exceptional spatial resolution but probes only extremely local regions, and precise control of *p*O_2_*via* electrochemical biasing is highly challenging. Environmental SEM enables tracking of the morphological evolution of composite electrodes; however, it lacks the capability for simultaneous surface-chemical analysis. NAP-XPS, in contrast, provides valuable chemical information on the surface but suffers from limited spatial resolution. It is therefore the objective of this work to employ an *in situ* method that enables simultaneous, surface-sensitive chemical analysis and spatially resolved morphological characterization, while controlling the effective *p*O_2_ of our material at operating temperatures.

Recently, the Electrochemical oXygen Activity ConTrol (EXACT) method was introduced, which allows for *in situ* surface analysis techniques in the absence of a gas phase under ultra-high vacuum conditions (UHV, *p*_tot_ ≈ 10^−12^ bar) while precise tuning *µ*_O_ – and equivalently the effective *p*O_2_.^39^ By employing an oxygen-ion buffering counter electrode, EXACT allows for well-defined electrochemical modulation of the working electrode's defect chemistry, which has already been successfully employed for XPS measurements on perovskite- and fluorite-type oxide electrodes.^39,40^ This development opens new possibilities for the *in situ* investigation of solid-state electrochemical systems through analysis techniques that rely on ultra-high vacuum conditions, enabling studies which would not be feasible using conventional gas-phase-based approaches.

In the present work, EXACT is for the first time implemented in an Auger–Meitner electron microscope (AMEM), enabling spatially resolved *in situ* monitoring of surface chemistry and morphology while systematically tuning the oxygen chemical potential, *i.e.*, the effective *p*O_2_. This approach provides direct access to polarization-induced defect formation and metal–support interactions, thereby allowing the morphological and chemical evolution of Ni/GDC model electrodes to be studied under precisely controlled, gas-free operating conditions.

In this study, we progress from simplified thin-films to near-realistic operating conditions. Our objectives in this regard are as follows:

• Selectively tuning the Ni/GDC interface energy without gas phase adsorbates – see Fig. 1a: for oxide-supported metal catalysts, it is often assumed that changes in adsorbates modify surface energies and thereby alter the wetting behaviour between metal and oxide.^41,42^ It is known that a hydroxyl or carbonate coverage strongly depends on electrochemical polarization and atmosphere.^43^ If the oxide is mixed conducting, as in the case of Ni/GDC, surface and interface energies are additionally altered by changes of the GDC defect chemistry (*i.e.* mainly Ce^3+^ concentration). This defect chemistry is strongly dependent on the effective *p*O_2_ of the gas phase. Consequently, changes in Ni/GDC wetting behaviour under different atmospheres can originate either from adsorbate effects or interface effects, and distinguishing between both is not straightforward. Hence, the first objective is to disentangle the effect due to *p*O_2_-induced GDC defect chemistry changes from adsorbate effects. To achieve this, it is important to omit possible changes in adsorbates, while changing the overpotential and, therefore, the effective *p*O_2_ of the GDC. This is realised by employing the recently developed EXACT technique^39^ which allows *µ*_O_ – and thus the effective *p*O_2_ – within GDC to be precisely tuned at high temperatures in UHV. This way, contributions of adsorbates to changes in interface energies are effectively absent, enabling us to isolate the impact of defect chemistry – and thus the interface component – on Ni/GDC dynamics in a first step.

**Fig. 1:**
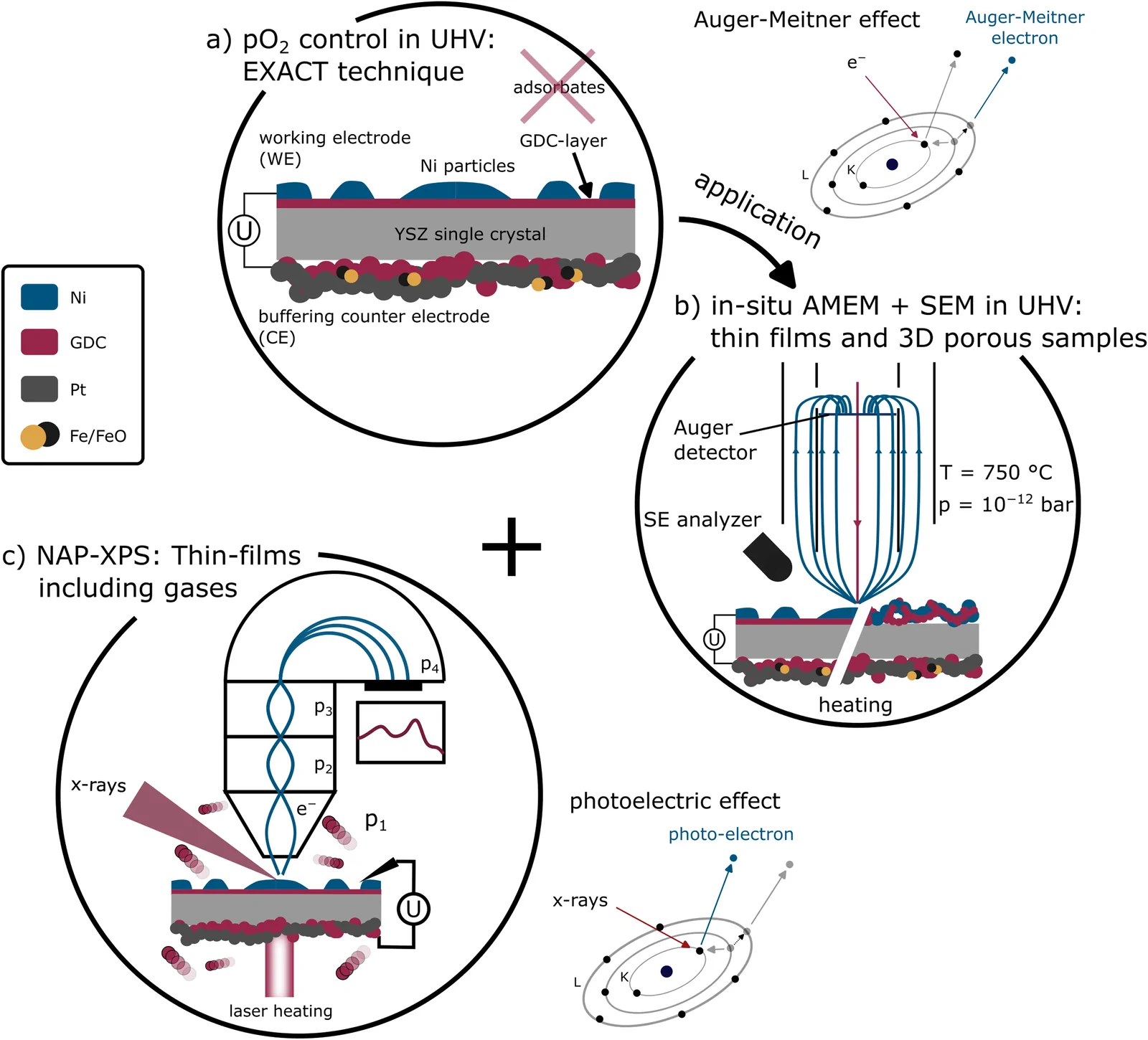
Graphical overview of the applied experimental principle for *in situ* monitoring the dynamics of Ni-dewetting on GDC at high temperatures under polarization. (a) Sketch of an electrochemical cell designed for oxygen activity control in UHV (EXACT technique). (b) Schematic setup of an Auger–Meitner electron microscope (AMEM) for *in situ* EXACT measurements on thin-films and 3D porous samples. (c) Schematic setup for *in situ* near-ambient pressure X-ray photoelectron spectroscopy (NAP-XPS) on thin-films.

• Probing Ni/GDC dynamics with novel *in situ* Auger–Meitner electron microscopy (AMEM) – see Fig. 1b: combining EXACT with AMEM enables *in situ* AMEM under well-defined GDC defect chemistry and nickel oxidation states at high temperatures. This approach makes it possible to follow the morphological changes in real time as the applied bias voltage – and thus, the defect chemistry of GDC – is varied. Furthermore, simultaneous surface-sensitive spectro-microscopy and electrochemical measurements are carried out, providing a direct link between interfacial chemistry and morphological dynamics. This constitutes the first pioneering application of Auger–Meitner electron microscopy as an *in situ* technique and, in combination with the EXACT method, establishes AMEM as a new *in situ* tool for SOC and catalysis research.

• Extending insights toward more application-relevant conditions: to enhance the practical relevance of the findings, experiments that more closely mimic real SOFC/SOEC conditions are carried out. Building on the UHV-based experiments, the Ni/GDC thin-film system is exposed to H_2_/H_2_O and CO/CO_2_ atmospheres, thereby electrochemically polarized, and analysed simultaneously by near ambient pressure X-ray photoelectron spectroscopy (NAP-XPS). In this way, the possible influence of gas-phase species on Ni/GDC dynamics was assessed and the transferability of the UHV-based insights to an operating Ni/GDC (electro-)catalyst was evaluated. In addition, real 3D porous electrodes originally developed for high-performance metal-supported SOCs^44^ were investigated using the EXACT-AMEM approach, showing that the effects identified in the thin-film model also occur in technologically relevant electrode architectures (see Fig. 1b).

The results unambiguously show that electrochemical redox-cycling acts as an essential trigger factor for morphology changes. We further demonstrate that this approach goes beyond method development by establishing Ni/GDC as a suitable model system and by increasing experimental realism through the transition from thin-film model electrodes to three-dimensional porous samples.

## 2 Experimental section

### 2.1 Sample preparation

An overview of the sample types used in this work is given in Fig. 2. For type 1 and 2 (Fig. 2a and b), the working electrode (WE) consisted of a thin-film-based Ni/GDC model electrode. In case of type 3 (Fig. 2c) the working electrode is a performance-optimized 3D porous Ni/GDC electrode, which was fabricated analogously to previous work.^6,44,45^ All three types of cells are equipped with a porous counter electrode (CE) that is based on Pt/GDC and contains a Fe/FeO phase mixture as an oxygen buffering system for *in situ* measurements in UHV.^39^

**Fig. 2:**
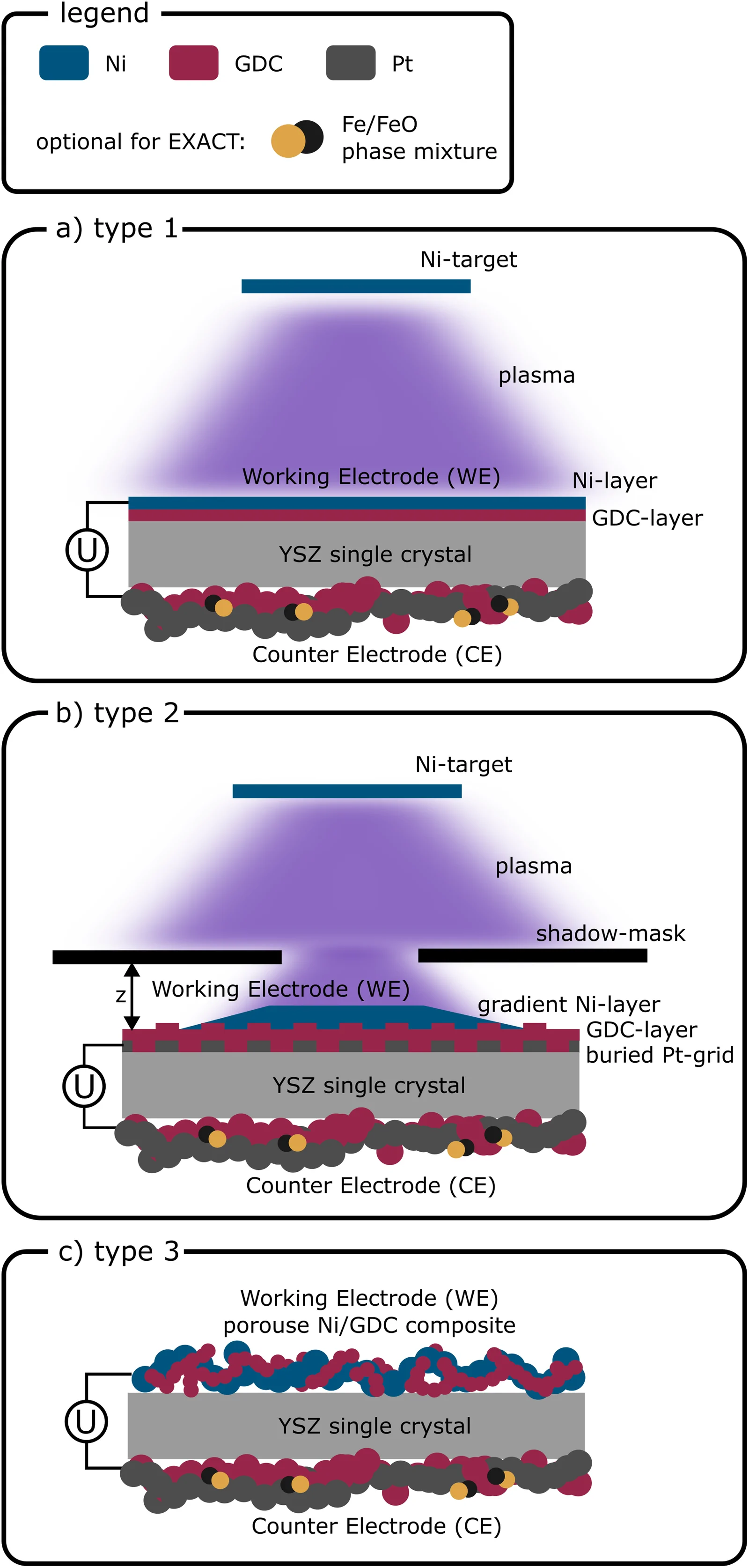
Schematic structure the different sample types used in this work. (a) Type 1, with the WE being a PLD layer of GDC directly deposited on YSZ. On top of that GDC layer, a sputtered Ni-layer is deposited, which also acts as CC. (b) Type 2, with WE being a PLD layer of GDC deposited atop a buried Pt-grid (CC). The Ni-layer deposited on the GDC exhibits a thickness gradient generated by a shadow mask. (c) Type 3, with the WE being a 3D porous Ni/GDC composite electrode *via* a powder-based route.

#### 2.1.1 Counter electrode

In the first step of model cell preparation, the porous oxygen buffering CE was applied on a YSZ single crystal (9.5 mol% Y_2_O_3_ in ZrO_2_, CrysTec GmbH, Germany), which served as the electrolyte. For the CE preparation, a GDC/Fe_2_O_3_ paste (80 wt% GDC, 20 wt% Fe_2_O_3_ – dispersed in terpineol) was spin-coated on the unpolished bottom side of the YSZ single crystal with 2400 rpm, and subsequently dried at 120 °C. After that, a Pt paste (Tanaka, Japan), which served as the electronic current collector, was brushed on top of the dried GDC/Fe_2_O_3_ layer. Subsequently, the CE was sintered at 1050 °C in air for 3 h.

After this sintering step in air, the iron in the CE was fully oxidized and in the state of Fe_2_O_3_. In order to pin the equivalent oxygen partial pressure of the CE (*p*O_2,CE_), however, a Fe/FeO phase mixture is necessary. This phase mixture was created in a CE conditioning step, which was carried out inside the AMEM setup. CE conditioning will be briefly explained in the Results section and is broken down in much more detail in ref. 39. Note that for NAP-XPS measurements, this CE conditioning step is not necessary, as the *p*O_2,CE_ is defined by the *p*O_2_ of the gas phase.

#### 2.1.2 Thin-film working electrodes

The thin-film WEs for both types 1 and 2 were prepared by Pulsed Laser Deposition (PLD) and magnetron sputtering. For type 1, a PLD layer of GDC (*ca.* 200 nm) was deposited directly on the polished side of the YSZ(100) single crystal. All depositions were carried out with a KrF Excimer Laser (*λ* = 248 nm, Lambda Physics, COMPex Pro 201) with a substrate to target distance of 6 mm, a substrate temperature of 600 °C and a *p*O_2_ of 0.04 mbar. This allows for well-defined epitaxial growth of the entire GDC film.^46,47^ On top of GDC, a dense Ni layer (*ca.* 200 nm) was deposited using magnetron sputtering. In case of this sample type, the Ni layer also acts as the electronic current collector (CC) for electrochemical experiments. Therefore, as the Ni film partially undergoes dewetting when heated up (see Fig. S2 and S3 in the SI), the film must be thick enough to remain percolating at high temperatures (*i.e.* minimum thickness of *ca.* 200 nm for measurements at 750 °C).

For type 2, a significantly thinner Ni film (*ca.* 100 nm) was deposited, designed to lose percolation when heated up to 750 °C. Since the Ni top layer in type 2 is thus no longer available as an electronic CC, it was necessary to apply an additional CC. This was achieved by fabricating a buried Ti/Pt grid below the GDC layer, on top of the polished side of the YSZ single crystal (15|5 µm mesh|strip width, 5 nm Ti below 100 nm Pt). The fabrication of this CC was carried out using magnetron sputtering, photolithography and ion-beam etching. On top of this CC, the GDC layer (*ca.* 200 nm) was grown by PLD. Subsequently, the thin Ni layer was sputter deposited, additionally using a shadow mask that was placed in a certain distance from the sample. This way, a gradient in the Ni layer thickness was achieved ranging from *ca.* 100 to 50 nm (see Fig. S5 and S6 in the SI). Upon heating this sample type, a lateral gradient of Ni particle sizes was introduced, which allowed us to select a Ni particle of ideal size for EXACT-AMEM.

The major difference between type 1 and 2 is thus that the Ni top layer remains percolating in case of type 1, whereas in type 2 it breaks up into individual particles, resembling the aspect ratio of real porous electrodes more closely. For type 1, the 200 nm thin Ni film initially dewetted only slightly when heated up to 750 °C (see Fig. S2 and S3 in the SI). In order to fine tune the degree of dewetting for our measurements, an electrochemical redox-cycling step (consecutive oxidation and reduction) was carried out. The reasons why this measure is suitable for specifically increasing the degree of Ni dewetting are discussed in detail in the results section.

#### 2.1.3 3D porous working electrodes

For sample type 3, the porous WE was prepared by applying a Ni/GDC paste formulated analogously to a Ni/GDC high-performance electrode described in previous work.^6,44,45^ Gadolinium-doped ceria (GDC = Gd_0.1_Ce_0.9_O_2−*δ*_, Treibacher Industry AG, lot n. EA35/12.1) and metallic nickel (Toho NF41) were the selected starting materials (details are provided in Table S1 and Fig. S1). The paste was prepared by mixing the Ni and GDC powders in 60 : 40 wt% proportion as follows: a pre-suspension was obtained by mixing 79 wt% of sonicated GDC powder, 1.03 wt% of dispersant Nuosperse FX9086 (Elementis Specialties, Inc. USA), 19.97 wt% of anhydrous terpineol solvent (Sigma-Aldrich) in a PET-bottle. Zirconia milling balls with diameters of 5 mm and 10 mm in ratio 1 : 1 were added to the bottle up to 2/3 of total volume and the pre-suspension was then mixed in a tumbler mixer for 48 hours at 67 rpm. In addition, a transport suspension was prepared in a hot mineral oil bath at 70 °C by mixing 85 wt% terpineol and the 15 wt% binder, *i.e.*, an ethyl cellulose powder (45 cP, medium chain length, Sigma-Aldrich). The final paste was obtained by slowly adding the nickel powder (32.8 wt%) to the GDC pre-suspension (27.7 wt%) and then combining this mixture with the transport suspension (22.5 wt%), and additional terpineol (16.9 wt%). Homogenization of the resulting Ni/GDC paste was obtained by four steps in an EKRA three-roll-mill.

This paste was spin-coated onto the electrolyte (5 × 5 mm YSZ single crystal) with a rotational speed of 6600 rpm. The resulting layer was dried at 120 °C and sintered under reducing conditions (2.5% H_2_ in Ar) at 1100 °C for 3 h. This was done subsequently to the preparation of the CE, which remained the same as for type 1 and 2 (explained in the sections above).

### 2.2 *In situ* Auger–Meitner electron microscopy

#### 2.2.1 Instrumentation and EXACT technique

*In situ* Auger–Meitner electron microscopy (AMEM) on the WEs of sample types 1–3 were conducted on a PHI 710 Scanning Auger Nanoprobe (Physical Electronics, USA) with minor modifications: an additional in-house built resistive heater was attached to the sample and connections for electrochemical analysis were established *via* Pt/Ir needles, which can be placed on the sample. Temperature control was established using the well-known temperature dependence of the oxide ion conductivity of the YSZ electrolyte single crystal.^48^ This conductivity was extracted from the sample's ohmic resistance (*i.e.*, the *x*-axis intercept in the Nyquist-plots) *via* electrochemical impedance spectroscopy (EIS).

For time-resolved SEM imaging (*U*_acc_ = 25 kV, *I*_emission_ = 1 nA), only the Everhart–Thornley-Detector (ETD) of the AMEM was used where roughly every 60 seconds, an image was taken. For spatially resolved Auger–Meitner electron spectroscopy (AMES), spectra were acquired at certain spots on the sample using the cylindrical analyser and the Auger–Meitner detector. For better signal/noise ratios, *I*_emission_ was set to 10 nA for spectroscopy and mappings. The latter was acquired using a 3-point method, where instead of measuring an entire spectrum, only the peak maxima and two points for the baseline are selected and measured. This dramatically reduces mapping times and can increase the signal/noise ratio. As both, mappings and time-resolved SEM imaging take longer times (up to 24 h), drift correction was necessary, which is a standard-feature of the used machine.

Please note that AMEM requires UHV (*i.e. p*_tot_ ≈ 10^−11^ bar), which means that virtually no gas atmosphere is present at the sample. *In situ* measurements on SOC electrodes – which commonly operate in gas atmosphere – become possible employing the so-called Electrochemical oXygen Activity ConTrol (EXACT) technique. For a comprehensive explanation of this method, please refer to ref. 39, as in the following paragraphs, this advanced *in situ* analysis technique can only be briefly summarized.

To understand the chosen EXACT approach, it is important to know that the rate determining step of mixed conducting SOC electrodes (such as GDC under reducing conditions) is located at the electrode surface, where oxygen exchange with the atmosphere occurs. Especially in case of thin-film electrodes, contributions from bulk transport or other resistances can be neglected. This holds in gas atmospheres,^49,50^ and is even more pronounced under UHV conditions, as almost no gas species are present at the electrode's surface. As a result, no significant exchange reactions between the atmosphere and the electrode material takes place. Consequently, also no faradaic current is observed when a SOC is polarized in UHV by applying a bias voltage between WE and CE.

For the 3D porous electrodes, the conditions are slightly different: in electrolysis the electrode polarization resistance is determined by both the ion transport within the GDC phase and the reaction resistance at the GDC surface, which are electrically connected in the form of a so-called transmission line circuit.^51^ This leads to the situation that with a gas atmosphere present, the electrochemical reaction takes place within a zone located next to the electrode/electrolyte interface, with the driving force for the reaction decaying from the interface into the 3D porous structure. The thickness of this electrochemically active zone is about 2*λ* with *λ* depending on the ionic conductivity of GDC *σ*_ion_ and the reaction resistance *R*_react_*via*eqn (1).^17^1
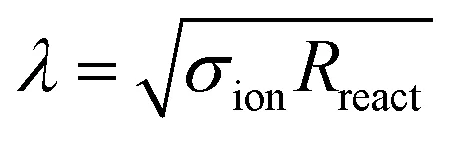


However, in UHV conditions, due to the absence of a faradaic DC current (high *R*_react_), there is no ion conduction overpotential within the electrode (high *R*_react_ → high *λ*). This means that for both thin-film and 3D porous electrodes, the oxygen stoichiometry and effective *p*O_2_ within the GDC phase is changed homogeneously upon electrochemical polarization as the electrochemically active layer becomes much larger than the actual thickness of our 3D porous Ni/GDC electrodes.

As the entire model cell is exposed to UHV, the absence of an exchange reaction also holds true for the CE. The central challenge of polarising a SOC in UHV is therefore establishing a stable reference potential at the CE so the chemical potential of oxygen at the WE is well-defined. In gas environments, the chemical potential of oxygen at the CE *µ*_O,CE_ is pinned by the oxygen partial pressure of the atmosphere. Under UHV this is not the case, so the chemical potential of oxygen at the CE would remain ill-defined. This was solved by incorporating a Fe/FeO phase mixture into the CE. As long as Fe and FeO coexist in equilibrium with each other, the chemical potential of oxygen stays well-defined and constant. The Fe/FeO buffer also equilibrates with the GDC/Pt CE, thereby stabilising *µ*_O,CE_ at a well-defined value with high oxygen buffering capacity. Electrochemically polarising the WE *versus* such an oxygen buffered CE by applying a voltage *U*_eq_ to the model cell, leads to a well-defined shift of *µ*_O,WE_ and the corresponding effective oxygen partial pressure (*i.e.* the oxygen activity) at the WE is defined by Nernst's equation – see eqn (2).2
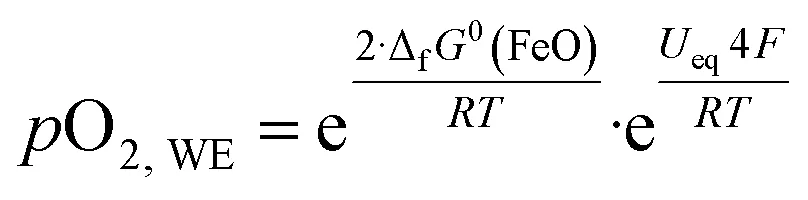


Therein Δ_f_*G*^0^(FeO) corresponds to the standard Gibbs free energy of the FeO formation, *T* is the absolute temperature, *R* the gas constant, and *F* is Faraday's constant. Hence, by controlling the applied bias voltage, *p*O_2_,_WE_ can be tuned in a well-defined manner. This approach enables the emulation of electrolysis conditions – *i.e.* same effective *p*O_2_ within GDC – without the presence of a gas phase.

#### 2.2.2 Counter electrode conditioning

The EXACT technique relies on a Fe/FeO equilibrium in the CE to provide a reliable reference *p*O_2_. As the CE was fabricated by sintering in air, at the beginning of the experiment, iron was in its fully oxidized form (Fe_2_O_3_). The Fe/FeO phase mixture was first established inside the AMEM setup by a CE conditioning step, in which a defined amount of O^2−^ ions was electrochemically pumped out of the CE. In order to avoid electrochemically altering the WE during this step, a third auxiliary electrode was used for this purpose, which essentially consisted of an electrically entirely separated segment of the WE. The CE conditioning was performed by applying an anodic bias to auxiliary *vs.* counter electrode in galvanostatic mode at 500 °C, which leads to the evolution of small amounts of O_2_ at the auxiliary electrode. The desired charge depends on the amount of Fe_2_O_3_ in the CE. In our case, approximately 0.7 C cm^−2^ was found to be an appropriate value for an effective CE conditioning. This procedure allowed conditioning of the CE while preserving a pristine WE segment for subsequent *in situ* AMEM measurements. After completion of the CE conditioning step, cyclic voltammetry was performed on the auxiliary *versus* the counter electrode. The appearance of the Ni/NiO transition at the expected potential (*vs.* Fe/FeO) confirmed the correct establishment of the Fe/FeO phase equilibrium at the CE and thus the stability of the CE potential.

#### 2.2.3 Coulometric titration of Ni/GDC working electrodes during EXACT-AMEM

When the WE is electrochemically polarized against the Fe/FeO-buffered CE and equilibrium conditions are established, the net current can be assumed to be zero. Upon abruptly changing the applied bias voltage, however, a significant capacitive current spike is observed. Besides electrostatic effects due to charging or discharging of electrochemical double layers at interfaces, which play only a minor role in the present case, this can be almost exclusively explained by the chemical capacitance of the Ni/GDC WE. This rather large capacitance mainly originates from two effects: first, from a change in the defect chemistry of GDC,^52^ as we change the equivalent *p*O_2_ at the WE, and therefore the equilibrium point defect concentrations of GDC. Second, from a phase transition, which is associated with a change in the oxygen content of one of the WE materials (*e.g.* a change from cubic fluorite-type GDC into another modification, or a Ni/NiO transition). In both cases essentially O^2−^ ions are electrochemically pumped in or out of the WE. Therefore, our model-type SOC operated in UHV essentially works like an oxygen-ion battery.^53^

To analyse these current spikes for the potentiostatic coulometry, upon changing the voltage in a stepwise manner, the current was measured with high time-resolution (50 m s^−1^). After roughly 60 seconds (for voltage steps of *ca.* 10 mV), the current reached a steady state close to zero and the current spike for this voltage step was integrated over time to calculate the charge, which is proportional to the amount of O^2−^ ions transferred in or out of the WE. This technique is referred to as a thin-film coulometric titration in literature,^54–56^ and was carried out simultaneously to *in situ* AMEM measurements in UHV.

### 2.3 Near ambient pressure X-ray photoelectron spectroscopy (NAP-XPS)

For dewetting experiments of Ni/GDC exposed to gas atmospheres, NAP-XPS was carried out using a lab-based setup equipped with a monochromatic Al K_α_ X-ray source (µFOCUS 500 NAP, SPECS, Germany). As the hemispherical analyser (PHOIBOS 150, SPECS, Germany) was positioned behind differentially pumped electrostatic lenses in this setup, measurements at *p*_tot_ = 0.5 mbar and higher are possible. For more details on this setup, please refer to ref. 57–59. This pressure is sufficient to electrochemically operate a model-type SOC in single-chamber configuration inside the NAP-XPS chamber. For this purpose, the sample holder is equipped with an IR laser for heating the sample from the backside and contact pins for electrical connection to the WE and CE. Temperature was controlled using a pyrometer, which was calibrated at low temperatures *via* the YSZ resistance determined by EIS, where cable and contact resistances are negligible compared to the ion transport resistance of the YSZ electrolyte.^57,59^

The NAP-XPS experiments were carried out in H_2_/H_2_O and CO/CO_2_ mixtures at total pressures of 0.4 and 0.5 mbar, respectively. Both gas atmospheres are directly relevant for solid oxide cells and were chosen because of their fundamentally different adsorption behaviour, which is expected to result in different surface energies of Ni and GDC under adsorbate coverage.^60^ The gas compositions were adjusted such that both correspond to the same effective *p*O_2_ of 3·10^−20^ bar at 750 °C. This equivalence arises from the equilibria in eqn (3) and (4), which define the equivalent oxygen chemical potential of the respective atmosphere. Matching the effective *p*O_2_ ensures that the concentration of point defects in GDC is the same in both environments, so that observed differences can be attributed to the distinct surface chemistry rather than to bulk defect chemistry. Please note that the NAP-XPS experiments are conducted in a single chamber, with both the WE and CE exposed to the same atmosphere. Electrochemical polarization drives complementary oxidation and reduction half-cell reactions at the two electrodes. However, these reactions balance each other, resulting in net zero gas-phase conversion. Therefore, the gas-phase composition and the associated *µ*_O_ remain constant during the measurement.3
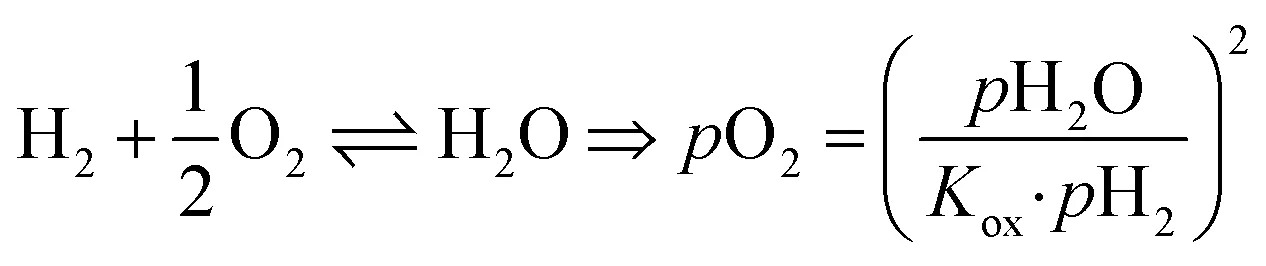
4
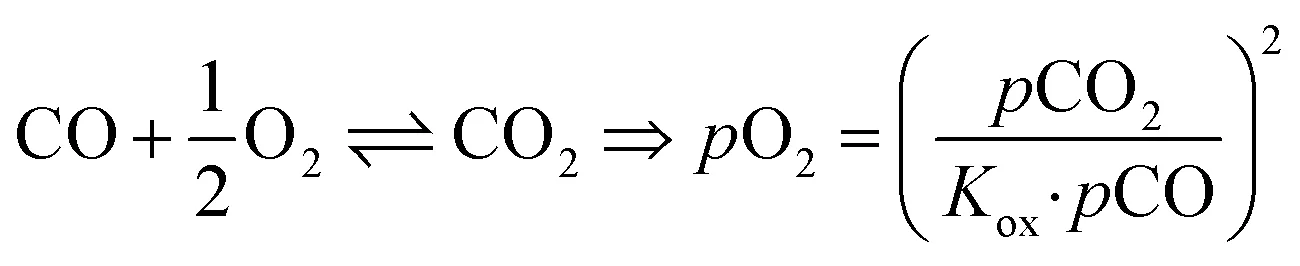


For NAP-XPS, model SOCs with a thin-film Ni/GDC working electrode were used (sample type 1, see Fig. 2a). The CE was fabricated in the same way as for *in situ* AMEM for reasons of simplicity. The iron phase, however, is not required under NAP conditions but does not interfere, since *µ*_O,CE_ is directly defined by *p*O_2,gas_. Consequently, under isothermal conditions, *p*O_2,WE_ is determined by *p*O_2,gas_ and the WE overpotential *η*_WE_. Because the CE is much faster than the WE and can thus be neglected, *η*_WE_ can be approximated by subtracting the ohmic voltage drop (*R*_Ω_·*I*_DC_) from the applied bias voltage as described in eqn (5).

5 *η*_WE_ ≈ *U*_set_ − *R*_Ω_·*I*_DC_

## 3 Results and discussions

### 3.1 *In situ* imaging of Ni/GDC dynamics on model-type thin-film electrodes

To study the effect of the oxygen activity (*i.e.*, the *p*O_2,WE_) on the wetting behaviour of Ni and GDC, *in situ* AMEM and *in situ* SEM were performed at 750 °C under UHV conditions (about 10^−12^ bar) using the EXACT technique. The details of this technique are explained in ref. 39 and briefly summarized in the Experimental section. This approach allows for simultaneous *in situ* AMEM/SEM measurements under polarization and electrochemical characterization techniques like coulometric titration and electrochemical impedance spectroscopy (EIS). For these experiments, sample types 1 and 2 with GDC and Ni thin-film WE and an oxygen ion buffering CE were used (see Experimental section for details). Thin-film XRD patterns of a pristine Ni/GDC sample are provided in the SI in Fig. S8 and confirm that GDC films are 100 oriented, while the Ni layers show 100 and 111 reflexes.

#### 3.1.1 Electrochemical redox processes in UHV

To validate that we can indeed control the oxygen activity of the WE by applying a bias with respect to the oxygen buffering CE (preparation described in Sections 2.1.1 and 2.2.2), the O^2−^ flux is measured as function of cell potential by three different methods: DC coulometry, electrochemical impedance spectroscopy (EIS), and cyclo-voltammetry (CV), in parallel to the SEM and AMEM measurements.

Sketches of the experimental program and the employed sample type are depicted in Fig. 3a and b. Raw data from DC coulometry is shown in Fig. 3c Clearly, after abruptly changing the voltage, a current spike is visible, which decays over a short period and approaches the comparatively small leak current (*I*_leak_). Results from EIS measurements are displayed in Fig. 3d.

**Fig. 3:**
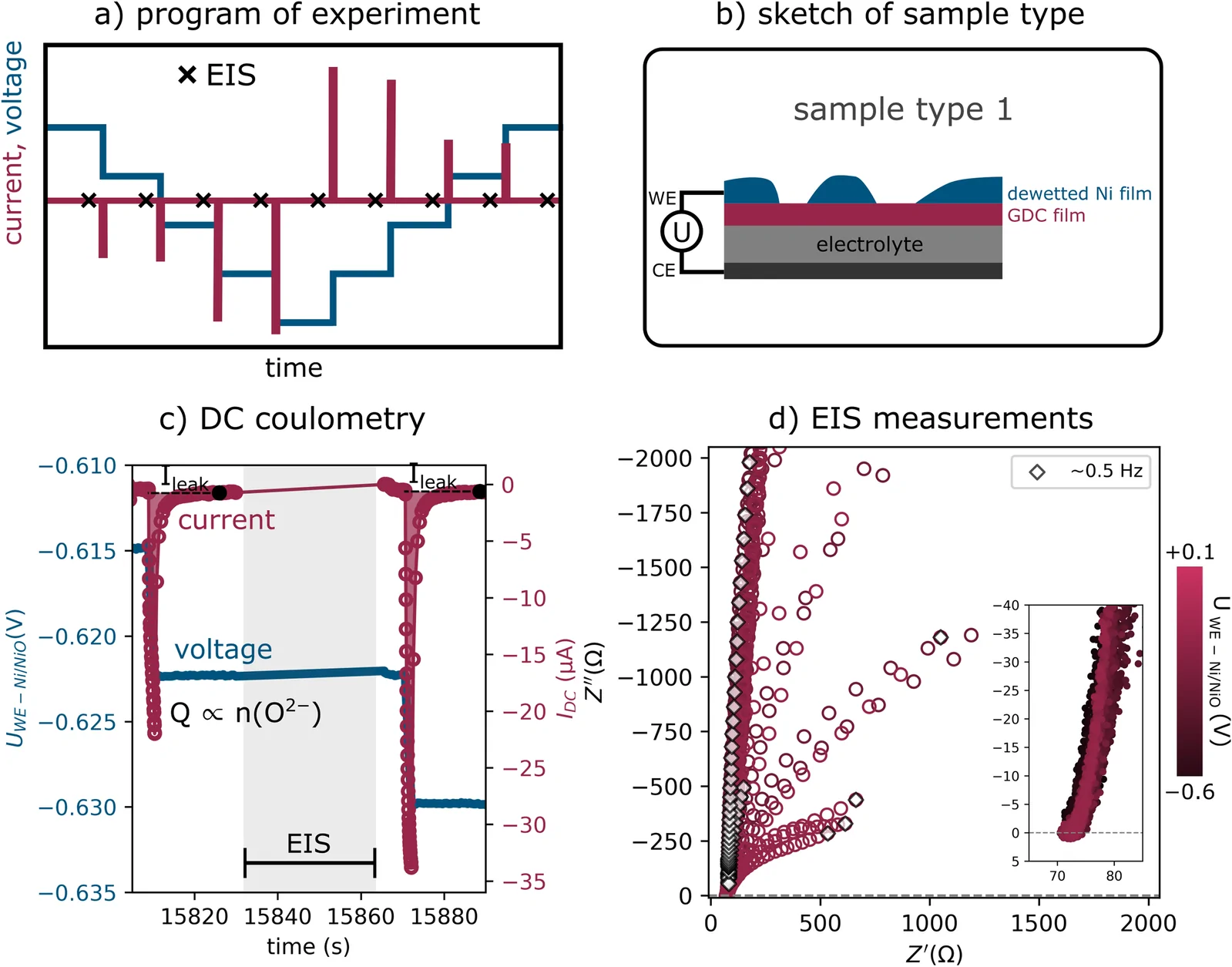
Electrochemical experiments conducted at 750 °C under UHV conditions: (a) sketch of the program of the experiment, (b) sketch of the thin-film sample type used for these measurements (type 1), (c) raw data from DC coulometry, (d) Nyquist-plots of raw data from EIS measurements.

Fig. 4a depicts the results from a DC Coulometric Titration (CT), where the oxygen content of our WE (2 − *δ* for GDC), is plotted against the voltage or equivalent *p*O_2_. It is derived from the resulting current spikes caused by an abrupt voltage change (*cf.*Fig. 3c) as follows: the spikes are integrated over time including a simple baseline correction, where the leak current *I*_leak_ is subtracted from the measured current *I* before integrating. From the resulting charge, the number of moles of oxide ions pumped in or out of GDC is obtained by eqn (6); *F* denotes Faraday's constant. The leak current mostly originated from a tiny electronic current through the YSZ electrolyte under strongly cathodic (*i.e.* reducing) polarizations.^61^*I*_leak_ was approximated by the current at the end of the voltage step (after decay of the current spike).6
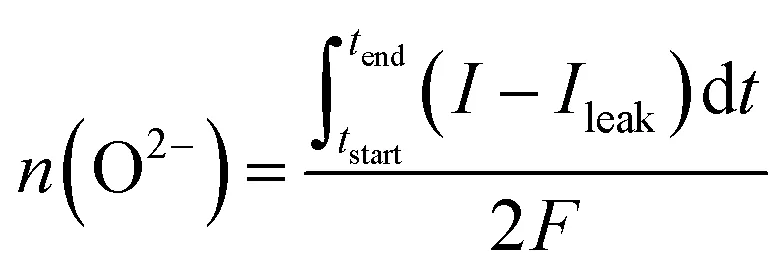


**Fig. 4:**
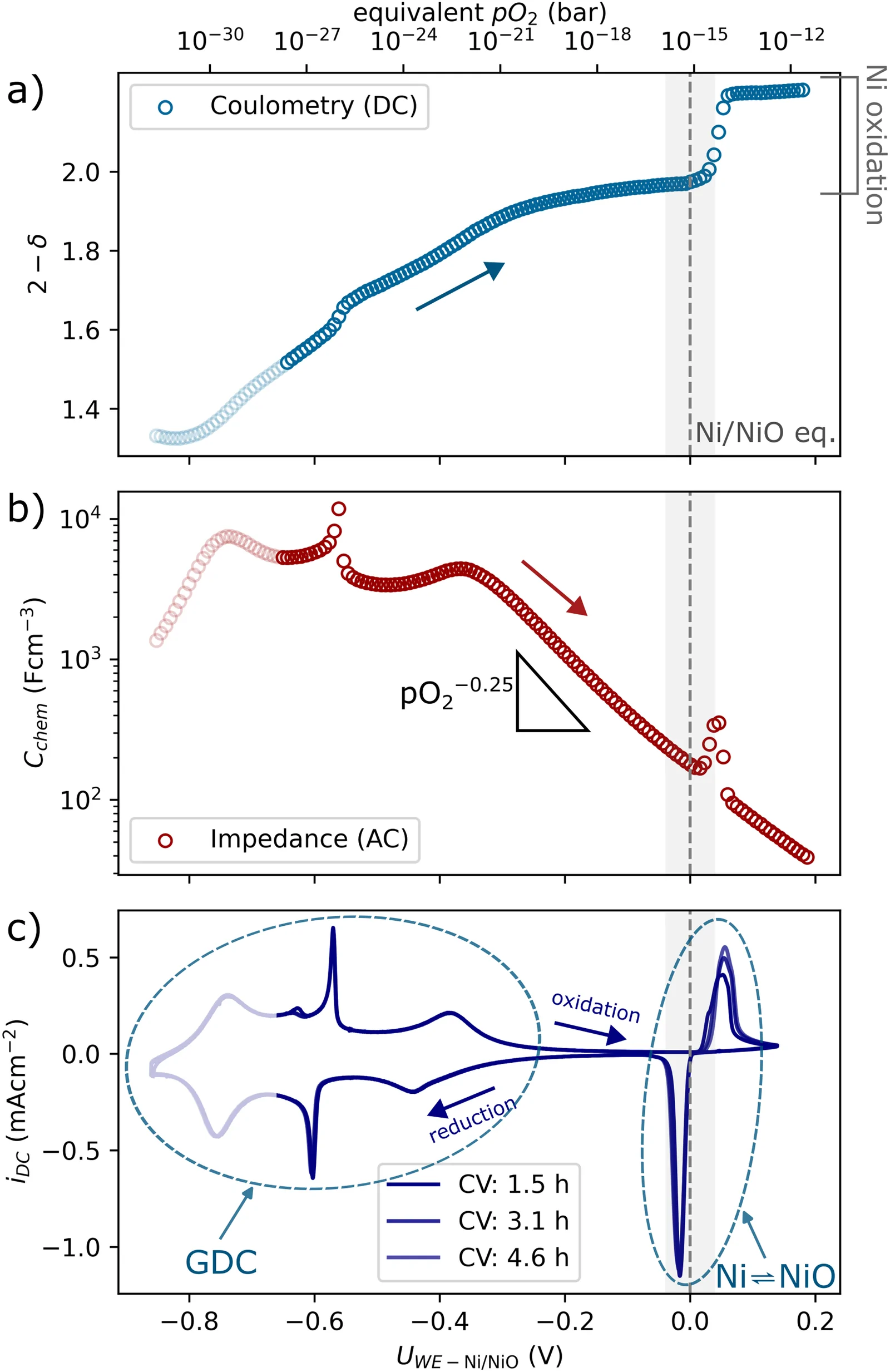
Electrochemical results obtained inside the AMEM on a cell with type 1 (thin-film sample Ni layer on GDC). Reduction of the GDC thin-film was determined by (a) the coulometric titration curve from DC measurements, (b) voltage dependent *C*_chem_ results from EIS measurements and (c) cyclovoltammetry.

At sufficiently low effective oxygen partial pressures at the WE (*p*O_2_ < 10^−12^ bar),^39^ neither a relevant oxygen evolution reaction nor an oxygen incorporation reaction occurs, because we operate under UHV conditions. The electrochemically pumped O^2−^ ions therefore solely alter the oxygen non-stoichiometry or phase composition of the WE. From *n*(O^2−^) (calculated from eqn (6)), the relative change in oxygen non-stoichiometry of the GDC thin-film can be derived, provided that the Ni/NiO equilibrium does not contribute to the chemical capacitance of the WE, which can be safely assumed below a *p*O_2_ of roughly 10^−15^ bar.

By looking at the *p*O_2_ region were only GDC reacts to the change in voltage, due to charge neutrality, a change in oxygen vacancy concentration 
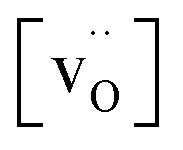
 – *i.e.* in *δ* – is coupled with a change in electron concentration as described by eqn (7). Note that the doping concentration 
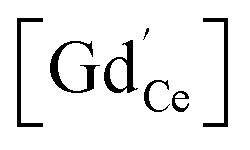
 remains constant (10% in Gd_0.1_Ce_0.9_O_2−*δ*_). Furthermore, electron holes are omitted in eqn (7), as their concentrations can safely be neglected under the strongly reducing conditions of the WE in the present experiments.^62^7
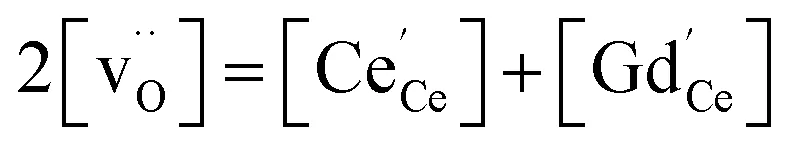


A change in 
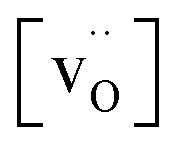
 (or *δ*) therefore directly alters the Ce^3+^ concentration 
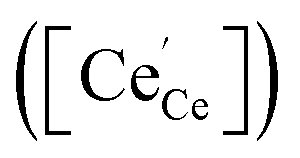
, which in turn determines the chemical capacitance (*C*_chem_) of our material.^63^ This is a differential capacitance that can either be derived by the slope of the coulometric titration curve (Fig. 4a), or directly by EIS measurements at each DC voltage step (details following below).

The oxygen non-stoichiometry *δ* in Gd_0.1_Ce_0.9_O_2−*δ*_ can be calculated *via*eqn (8). Therein *δ*_0_ corresponds to oxygen non-stoichiometry at the start of the experiment, which is assumed to be 0.05 due to 10% Gd^3+^ concentration, and *n*_GDC,film_ the number of moles of GDC within the entire film.8
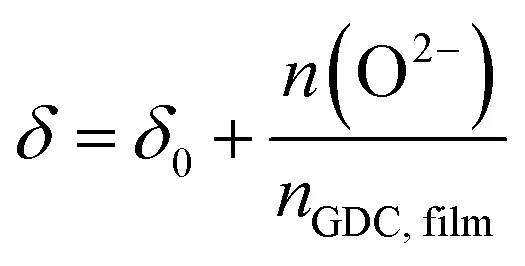


Once the current spike during coulometry had decayed – *i.e.*, after reaching a steady state – EIS measurements were performed while maintaining a constant DC bias. The resulting Nyquist-plots are depicted in Fig. 3d. These measurements were performed on the same sample as was used for recording the coulometric titration data shown in Fig. 4a, even within the same experimental run.

EIS data from Fig. 3d can be parameterised in very good approximation using a simple *R*_Ω_–*R*_ode_‖*C* equivalent circuit. Exemplary fitting results are shown in the SI in Fig. S7. The obtained parameters, *R*_Ω_, *R*_ode_, and *C* correspond to the ohmic resistance of the cell (mainly resistance of ion conduction in YSZ), the surface exchange resistance of the WE (which is very high under UHV conditions), and the measured capacitance originating from the chemical capacitance (*C*_chem_) of the WE, respectively. As emphasized by the zoomed inset, the ohmic serial resistance *R*_Ω_ is not altered at all when a bias voltage is applied and stays constant throughout the entire experiment. This suggests that the Ni film does not fully dewet within our measurement, but rather remains percolating and thus the entire electrode stays electrochemically active.

The chemical capacitance (*C*_chem_) is a bulk property of our GDC WE, and is straightforwardly obtained by normalising the obtained fit parameter *C* to the volume of our GDC thin-film (*V*_tf_ ≈ 2·10^−6^ cm^3^). Fig. 4b depicts the *C*_chem_ values of the WE as a function of *U*_WE–Ni/NiO_ and effective *p*O_2_.

Fig. 4c depicts three consecutive CV curves (*i.e.* DC current plotted *versus U*_WE–Ni/NiO_) with a linear scan rate of 5 mV s^−1^ taken in a separate experiment with a nominal identical sample. The final time of each CV cycle with respect to the start of the experiment is depicted in the legend.

It is important to note that for all plots shown here, the voltage at the WE (*U*_WE_) is referenced to the Ni/NiO equilibrium potential (*U*_Ni/NiO_) as described by eqn (9). *U*_Ni/NiO_ in turn was determined by cyclovoltammetry.

9 *U*_WE–Ni/NiO_ = *U*_WE_ − *U*_Ni/NiO_

The effective oxygen partial pressure *p*O_2,WE_, *i.e.*, the oxygen activity of GDC, is calculated using eqn (10), referring to the corresponding effective oxygen partial pressure of the Ni/NiO transition from literature (*p*O_2,Ni/NiO_ = 9.3·10^−16^ bar at 750 °C (ref. 64)).10
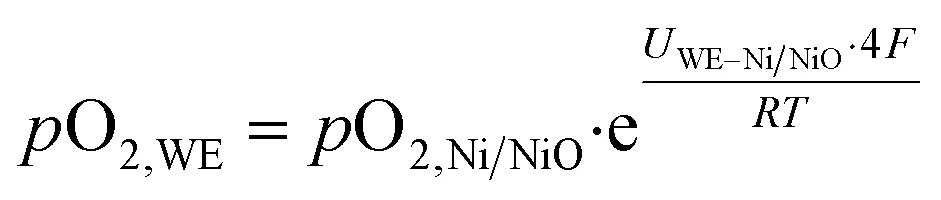


It should be emphasized that eqn (10) is fully equivalent to eqn (2), as both expressions result in the same value for the effective oxygen partial pressure. However, referencing to the Ni/NiO equilibrium offers a more practical and intuitively accessible link to the redox processes in the Ni/GDC system. For this reason, this notation is used consistently from this point onward.

Since the results of the three methodologically different measurements that are depicted in Fig. 4 contain information about the same electrochemical processes in the WE, they shall be discussed collectively in the following:

All graphs in Fig. 4 display the Ni/NiO phase transition at the thermodynamically expected value at a *p*O_2_ of 9.3·10^−16^ bar at 750 °C,^64^ as indicated by the dashed grey line including an uncertainty of ±40 mV. In the CT curve in Fig. 4a, the Ni/NiO transition becomes evident by a steep section in the CT curve above O_1.95_. Please note that an oxygen content of O_1.95_ cannot be exceeded due to the acceptor doping of the GDC layer. A nominal increase beyond this level indicates the oxidation of Ni to NiO. By a simple calculation from *n*(O^2−^) in this region, we can approximate the thickness of the formed NiO layer at the GDC interface to be roughly 8 nm. The Ni/NiO transition is also visible as a peak-like feature in the *C*_chem_ and CV curves in Fig. 4b and c. This confirms that upon changing the voltage, the oxygen chemical potential (*i.e.* the effective *p*O_2_) at the WE can indeed be precisely tuned.

The slope of log(*C*_chem_) *versus* log(*p*O_2_) in Fig. 4b in the moderately reducing regime (*U*_WE–Ni/NiO_ = −300 mV to 0 mV) is close to the theoretically expected value of −0.25.^65^ Since the chemical capacitance is proportional to the concentration of the minority defect species of a mixed conductor and the defect model of GDC describes n-type electronic point defects as minority charge carriers with precisely this *p*O_2_^−0.25^ dependence in this *p*O_2_ range, we can conclude that by electrochemically polarising the WE, we can precisely modulate the oxygen activity and thus the Ce^3+^ concentration in GDC.^52,66^ This is confirmed by the CT curve in Fig. 4a, as the oxygen content in GDC is altered from O_1.95_ to roughly O_1.80_.

Upon applying more and more cathodic bias, the oxygen vacancy concentration in GDC gradually increases, with a discontinuity (steep section) at *U*_WE–Ni/NiO_ of roughly −550 mV, and an oxygen non-stoichiometry of roughly 1.7 (see Fig. 4a). In addition, we observe sharp spikes in the *C*_chem_ and CV curves (Fig. 4b and c) at the exact same potential as the steep feature in Fig. 4a. This may correspond to a phase transition of the cubic fluorite structure of GDC to a bixbyite-like structure (*i.e.* Ce_2_O_3_ structure), which is reported in literature to become already stable at oxygen contents below 1.75.^67,68^ Unfortunately, without clear phase information, no unambiguous evidence of such a phase transformation can be provided from the available data, but since both the effective *p*O_2_ range and oxygen content of GDC are consistent with a transformation to a bixbyite-type structure, we consider this to be the most plausible explanation for this feature. For further validation, however, *in situ* XRD studies would be necessary.

Please note that at voltages more cathodic than about −650 mV *vs.* Ni/NiO all displayed data points are greyed out, as at *p*O_2_ values within the order of 10^−30^ bar, we operate under extremely reducing conditions where the YSZ electrolyte becomes a partial electron conductor.^61,69^ Therefore, the values for 2 − *δ* are significantly less accurate in this regime because of non-negligible electronic leak currents.

What is also worth emphasising is that the *C*_chem_ curve in Fig. 4b qualitatively matches the first derivative of the CT curve in Fig. 4a with respect to voltage. This relationship makes perfectly sense, since the CT curve depicts a charge (*Q*) *vs.* voltage (*U*) relation and the differential capacitance is defined as d*Q*/d*U*. In fact, the *C*_chem_ curve is not derived by simply calculating the derivative of the CT curve, but by measuring the differential capacitance using impedance spectroscopy, which proves that the two methods are consistent.

Moreover, Fig. 4b and c are also in good agreement, since peaks in CV curves also depict capacitances, which are chemical capacitances in this case. Notably, the part of the CV curve measured in “reducing” direction is mirrored to negative currents, as usual in CV curves. Also, the sharp peaks in the CV curves measured in “reducing” and “oxidising” direction are shifted slightly to more cathodic and anodic values, respectively. These shifts are a common feature in CV measurements, which can be assigned to the applied linear voltage ramp (5 mV s^−1^) that was used to record these curves. Since the voltage is continuously swept at a finite rate, the overpotential dropping in the cell causes the anodic and cathodic peaks to appear at potentials shifted from their equilibrium values. For the redox peaks in GDC, this shift is in large parts attributed to the ohmic resistance of the electrolyte. The Ni/NiO transition has a higher shift, due to its phase transition kinetics causing an additional polarization resistance.

To summarize, from electrical data discussed above, we can determine the bulk non-stoichiometry properties of our electrodes. In the following chapter, *in situ* (EXACT) AMEM and SEM results will be presented to investigate the dynamic changes of the Ni/GDC's surface chemistry and morphology upon changing the voltage.

#### 3.1.2 Time-resolved EXACT-SEM imaging of thin-films in UHV

To study the isolated effect of the oxygen activity of GDC on the morphology dynamics of Ni/GDC electrodes, EXACT-SEM imaging under varying voltages was conducted under UHV conditions. For these measurements, an AMEM microscope was used as described in the Experimental section, but only the ETD detector was employed for time-resolved measurements (mostly morphological information).

For the following measurements, we are looking at two slightly different sample types (see Fig. 2): for type 1, GDC was directly deposited on top of the YSZ. The Ni layer was grown thick enough to stay percolating since it also acts as the current collector (CC) and SEM imaging was carried out in top-view. Sample type 2 includes a buried Pt-grid below the GDC layer. This allows to investigate much lower film thicknesses of Ni, which lead to smaller isolated droplets when heated up to 750 °C. Moreover, these smaller particles were analysed from a 50° tilt angle to get a better view of the wettability of these particles.

In Fig. 5 and 6 the corresponding EXACT-SEM images at 750 °C for type 1 (topview) and type 2 (50° tilt) are depicted, where each potential step was kept constant for a certain time (*ca.* 0.5 h for type 1 and *ca.* 1 h for type 2). The electrochemical voltage programs (*U*_WE–Ni/NiO_) are plotted against time in Fig. 5a and 6a, respectively, where grey markings indicate the voltage/time coordinates for the depicted SEM images in the following subfigures. The dashed horizontal red lines in Fig. 5a and 6a indicate the Ni/NiO equilibrium potential, which is derived from CV curves for each type. The right *y*-axis in Fig. 5a displays the evolution of Ni coverages against time, as calculated from image segmentation.

**Fig. 5:**
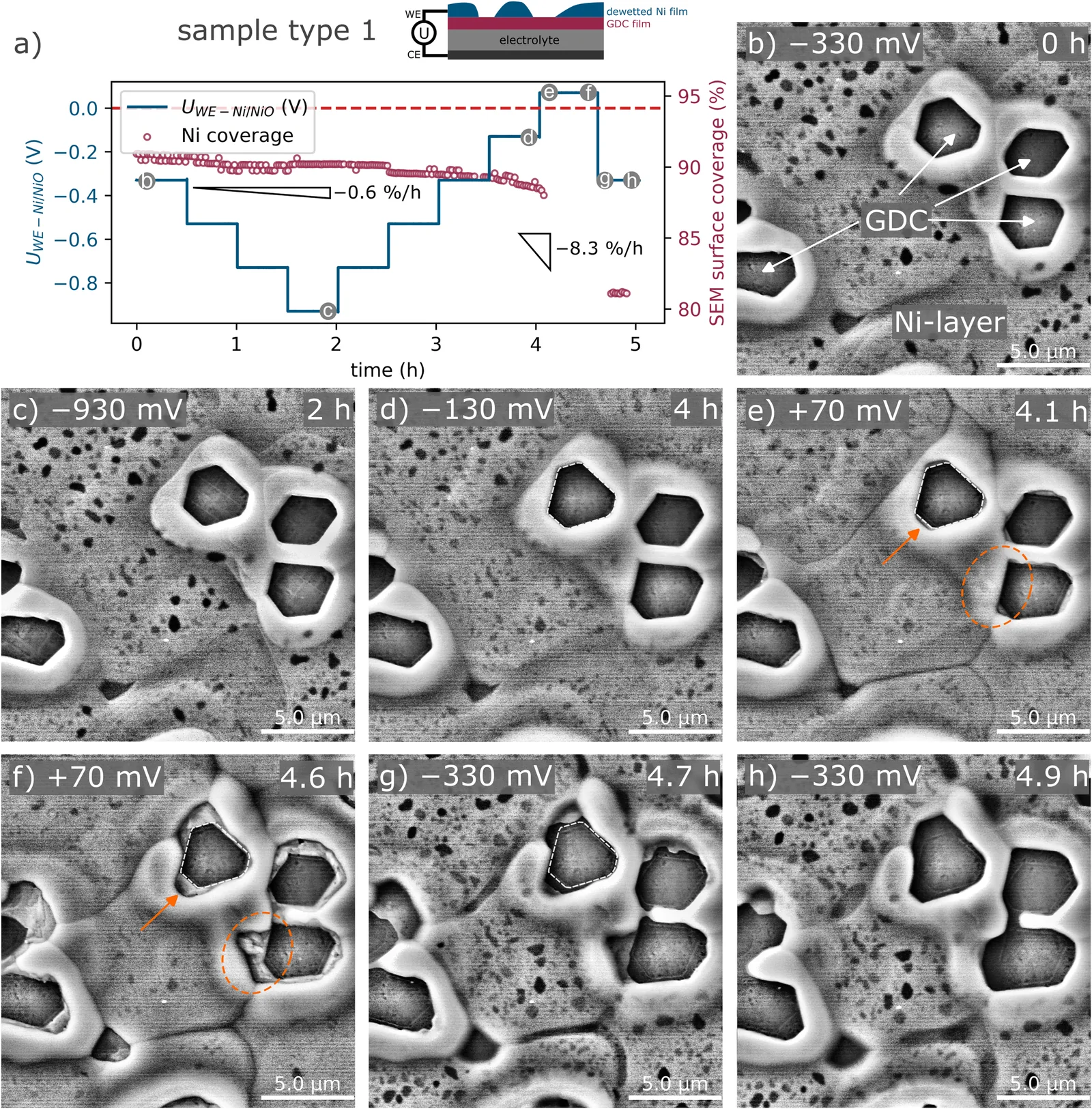
(a) *U*_WE–Ni/NiO_ plotted against time for EXACT SEM imaging including calculated Ni surface coverage: the red dashed line indicates the Ni/NiO transition potential derived from cyclovoltammetry, grey markings indicate voltage/time coordinates for SEM images. (b–h) EXACT SEM images in at 750 °C under varying voltages *vs.* Ni/NiO for geometry 1 (topview). Orange arrows and orange ellipses indicate regions of Ni oxidation.

**Fig. 6:**
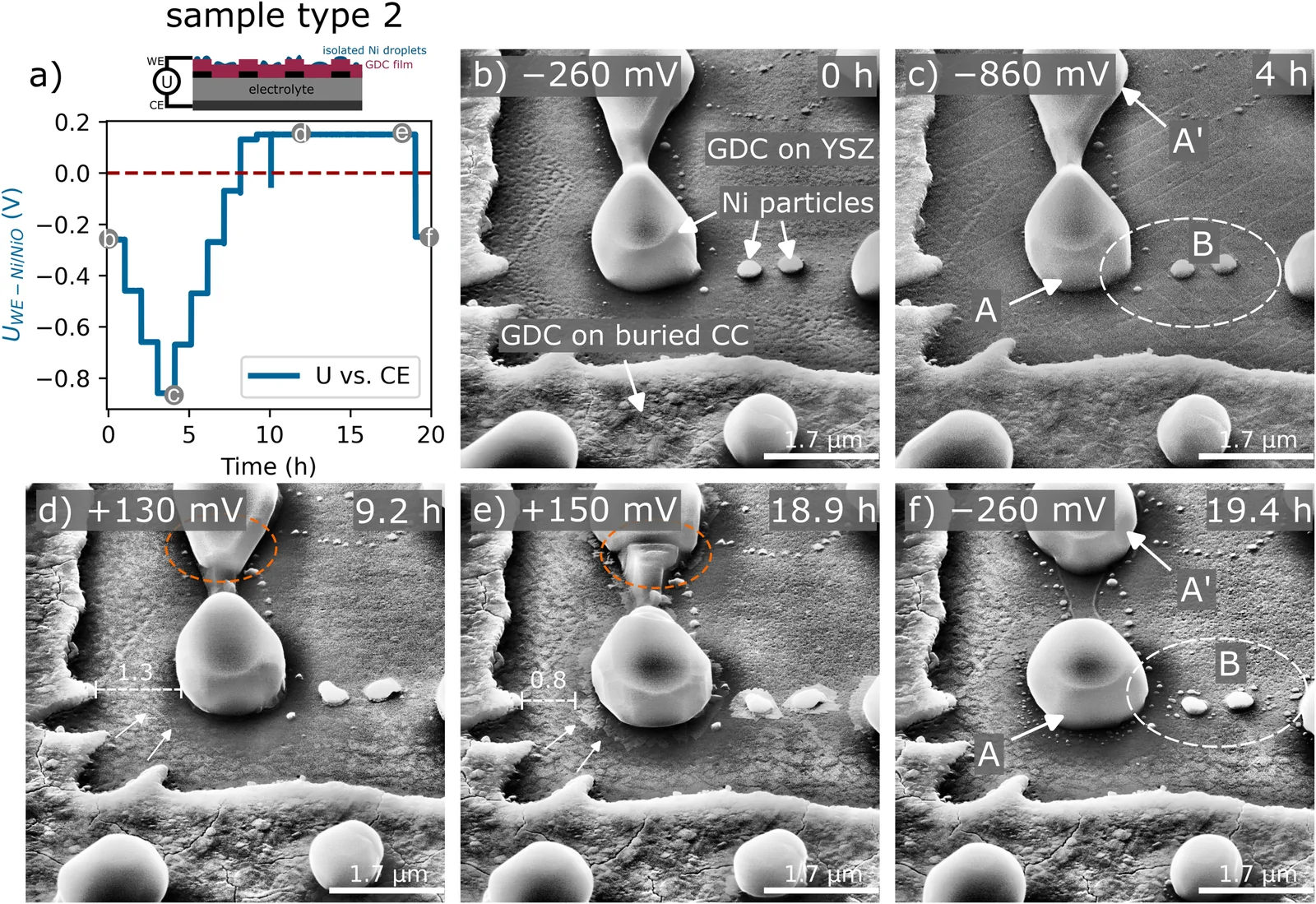
(a) *U*_WE–Ni/NiO_ plotted against time for EXACT SEM imaging, the red dashed line indicates the Ni/NiO transition potential derived from cyclovoltammetry, grey markings indicate voltage/time coordinates for SEM images. (b–f) EXACT SEM images at 750 °C under varying voltages *vs.* Ni/NiO for geometry 2 (50° tilt). Orange ellipses indicate regions of Ni oxidation.

Here, we observe the morphological changes upon tuning the equivalent *p*O_2_ of the WE, more specifically, of the GDC layer, solely by applying an electrochemical bias without the presence of a gas phase (*i.e.* in UHV, *p*_tot_ = 10^−12^ bar).

In Fig. 5 (type 1), we see a Ni-layer on top of an underlying GDC layer, which had been grown on the YSZ(100) single crystal directly (see Fig. 2a). The Ni film exhibits a number of pinholes, through which the GDC layer is visible. In addition to the larger pinholes, also smaller dark stains are visible. These are probably cavities caused by a previously executed redox-cycling step, which was necessary to create the desired pinholes.

Fig. 6 (type 2) depicts rather isolated Ni droplets on top of the GDC layer. Some of the Ni particles are labelled as A, A′ and B. In this case, also two distinct regions of GDC can be distinguished: one where GDC was grown directly on YSZ(100) – *i.e.* between the Pt current collector strips – and another one where it is grown on Pt, which leads to a GDC film growth were multiple crystal orientations occur.

For better time resolution of the morphological changes, the authors would like to encourage the reader to look at the time-resolved EXACT-SEM videos, which are provided as SI (see files named 01_type1_staircase.mp4 and 02_type2_staircase.mp4). The different effects of the experiments in Fig. 5 and 6 shall be described in the following section.

##### 3.1.2.1 Morphological changes while remaining more reducing than the Ni oxidation potential

The morphology evolution of the samples under reducing conditions (below the Ni/NiO transition potential) is depicted in Fig. 5b–d, 6b and c for sample type 1 and type 2, respectively. Within the timeframe of the first step at a constant potential no substantial morphology changes are observable. Also, when the bias is changed in a stepwise manner towards stronger cathodic polarization (after a duration of 2 h and 4 h for type 1 and 2, respectively), no substantial changes in the Ni morphology are observed.

Interestingly, under strongly reducing conditions (−930 mV in Fig. 5c and −860 mV in Fig. 6c), orthogonally oriented stripe-like features in the GDC layer become visible, which again disappear once we return to moderate reducing conditions (Fig. 5d). Regarding Ni-dewetting, however, still no significant changes are observable within the first 4 (type 1) or 8 hours (type 2) of the experiment under reducing conditions. Investigating the exact origins of these lines in the GDC layer is beyond the scope of this work. However, it is likely that these features are related to the chemical expansion^70,71^ or a phase transition^67,68^ of GDC under strongly reducing conditions.

We therefore conclude that upon changing the oxygen activity in our GDC substrate under reducing conditions (below the Ni/NiO threshold), the wettability of Ni on GDC is not changed effectively within the measurement's timeframe.

##### 3.1.2.2 Morphological changes upon exceeding the Ni oxidation potential

Fig. 5e, f, 6d and e show snapshots of the morphology evolution under sufficiently high (*i.e.* positive) voltages that allow for Ni oxidation. Without going into too much detail about the spectroscopic results at this point – these will be discussed in the following chapters – it should be noted here that the additional phase appearing between Ni and GDC corresponds to NiO. Upon NiO formation, strong changes in morphology are clearly visible within a short timeframe.

In Fig. 5e (type 1), additional grain boundaries/cracks are appearing on top of the Ni-layer's surface. At the TPB between GDC, Ni, and vacuum, the *in situ* formation of an additional phase – the NiO phase (see spectroscopy chapter) – is clearly visible. Over the course of 30 minutes, more and more NiO becomes visible (indicated by orange arrows and orange ellipses in Fig. 5e andf) as the metallic Ni atop NiO retracts from the TPB.

Fig. 6d and e (type 2) shows a very similar behaviour. However, for these smaller and isolated particles, the oxidation of Ni occurs in a more pronounced manner. The morphology of the thin connecting region between particles A and A′ changes significantly, appearing to be fully oxidized. Again, the metallic Ni atop the newly formed NiO migrates away from the connection region towards the outer particle A′ (see orange ellipse in Fig. 6d ande).

The observed Ni oxidation is in accordance with electrochemical data (see Fig. 4). The Ni oxidation occurs right above the Ni/NiO threshold confirming the effective electrochemical polarization of GDC. Since in this setup, virtually no gas phase is present, the oxidation reaction can only be facilitated by increasing the *p*O_2,WE_ using an electrochemical driving force. Furthermore, we observe that the Ni film is not fully oxidized, which reaffirms the slow rate of the Ni oxidation reaction very likely caused by the slow oxygen transport through NiO due to the relatively low diffusion coefficient of Ni vacancies in NiO.^72–74^

The morphologic evolution demonstrates severe changes under redox-cycling conditions as the Ni coverage on GDC dramatically decreases. This is already clearly visible on a qualitative basis in Fig. 5 and the corresponding EXACT-SEM video in the SI. To quantify the different rates of dewetting under reducing and oxidising conditions, we extract the area fractions of Ni and GDC by using an image segmentation algorithm. For details on the used watershed segmentation, refer to the SI (Fig. S9–S11). In Fig. 5a, the calculated Ni surface coverage is plotted against time on the right *y*-axis. It is clearly visible that during the first 4 h of experiment, the degree of dewetting is rather slow with a slope of −0.6%/h (% Ni coverage per hour). Upon redox-cycling, the dewetting rate increases to −8.3%/h, which demonstrates that the dewetting rate is more than 10 times faster under redox-cycling than under strongly reducing conditions. Please note that these rates are specific to the present sample geometry and operation conditions, and shall not be viewed as universal kinetic metrics. Rather, they serve as an internally consistent, semi-quantitative comparison between redox conditions within the same *in situ* experiment.

As the Ni oxidation is facilitated at the Ni/GDC interface, not the interface between Ni and GDC, but the interface between Ni and NiO plays the important role. Apparently, Ni-dewetting is much faster on NiO than on GDC. The reason for this effect may be complex and manyfold: first, the interface energy between Ni and NiO could be higher compared to the interface energy between Ni and GDC (*W*_int,Ni|GDC_ < *W*_int,Ni|NiO_). This would lead to an increased driving force for dewetting. Second, the diffusivity of Ni on NiO could be higher than on GDC, which could lead to faster dewetting kinetics. Third, the chemical expansion and contraction upon nickel oxidation and reduction may generate mechanical stresses which could also play a major role in accelerating dewetting. For instance, in-plane stress gradients resulting from interface expansion upon NiO formation may influence the stability of our metal thin-film.^75^ In such a case, the relaxation of the corresponding strain energy might be the driving force for the observed accelerated dewetting. Furthermore, cyclic stresses due to redox-cycling may induce surface roughening and influence surface diffusion. While several additional mechanisms may also contribute to the observed behaviour, the available data does not allow clear identification of the dominant process and a detailed mechanistic investigation is beyond the scope of the present work.

By prolonged anodic polarization above the Ni/NiO threshold, an additional effect can be observed (Fig. 6d ande): after several hours at oxidising conditions a thin layer of NiO seems to cover parts of the adjacent GDC surface, as illustrated by the dashed scale bars and white arrows in Fig. 6d ande. This spread of NiO on GDC indicates a much stronger interaction between the two oxides compared to the metal–support interaction between Ni and GDC (*W*_int,NiO|GDC_ < *W*_int,Ni|GDC_). Similar effects on the overgrowth of NiO on oxide supports have already been found in literature using *operando* NAP-XPS^72^ and post-mortem SEM.^76^ From the extent of the NiO spread in Fig. 6d ande, we can estimate the surface diffusivity of NiO on GDC at 750 °C using eqn (11).11
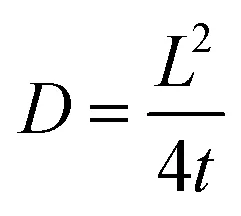


Therein *D*, *L*, and *t* are the diffusion coefficient, the diffusion length (assuming an error function having decayed to 0.16), and the diffusion time, respectively. Plugging the distance travelled (0.5 µm) and the time difference between recording of Fig. 6d ande (9.7 h) into eqn (11) leads to a surface diffusion coefficient of NiO on GDC of 1.8·10^−14^ cm^2^ s^−1^ at 750 °C. This value is very close to the cation self-diffusion coefficient of Ni^2+^ in NiO of roughly 2·10^−14^ cm^2^ s^−1^ known from literature at these temperatures,^77,78^ which is consistent with our interpretation that nickel in its oxidised form is indeed the diffusing species.

After the oxidation, the applied voltage was abruptly set back to reducing conditions under which NiO is no longer stable (Fig. 5g, h and 6f). A sudden change in morphology is visible within seconds. In case of both sample types, the *in situ* grown NiO disappears almost immediately and the roundish shapes of the initial morphology of metallic Ni is recovered. This is most pronounced in Fig. 6e andf, as instead of the ultra-thin NiO layers (white arrows and region B) – which covered large areas of the GDC surface upon oxidation – droplet-like metallic nanoparticles form immediately upon reduction. This disintegration of the previously covering NiO layer into extremely small Ni metal particles with much less interface area to GDC may again be explained by the interfacial energy between Ni and GDC being much higher compared to NiO and GDC. A very similar mechanism has already been proposed in literature.^76^ However, this phenomenon has never been investigated thoroughly using *in situ* measurements until now.

Finally, after consecutive oxidation and reduction (redox-cycling), the TPB of the re-reduced Ni layer does not retain its original position. Ni now covers much less of the GDC when compared with the morphology before redox-cycling (compare Fig. 5d and h). For type 2, this is even more evident as regions A and A′ are now completely isolated, almost half-spherical particles compared to the initial morphology (compare Fig. 6c and f). Additionally, the smaller and dark stains re-appear. These are most likely cavities located at the Ni/GDC interface that are caused by redox-cycling, as NiO has a higher molar volume than Ni.

In summary, time-resolved EXACT-SEM imaging at 750 °C reveals that Ni dewetting on GDC depends strongly on the applied redox conditions. Below the Ni/NiO threshold, where Ni remains metallic, dewetting proceeds slowly and steadily, independent of the applied polarization. Redox-cycling, on the other hand, accelerates this dewetting by more than an order of magnitude. Above the Ni/NiO threshold, oxidation of Ni drives strong morphological change, as the Ni/GDC interface is replaced by the Ni/NiO interface. Upon re-reduction, NiO converts back to Ni almost instantaneously, but the resulting Ni/GDC morphology remains irreversibly altered, with Ni coverage staying strongly decreased.

In the next chapter, we will take a closer look at the surface chemistry of the Ni/GDC thin-films by means of EXACT-Auger–Meitner electron spectroscopy and microscopy (AMES/AMEM).

#### 3.1.3 *In situ* Auger–Meitner electron spectroscopy and microscopy

After looking at the dynamics of Ni/GDC interactions, a chemical assignment of the present phases observed in SEM images is required. For this task, spatially resolved surface sensitive EXACT-AMES/AMEM at operating temperatures (750 °C) is employed.

For sample type 1, Auger–Meitner electron spectroscopy and microscopy (AMES/AMEM) measurements were performed on the same sample (and position) directly after the time-resolved SEM measurements. First, spatially resolved AMES was carried out. Fig. 7a depicts the corresponding SEM images with markings for regions A and B, indicating the locations where the spectra were acquired. Fig. 7b shows the obtained AMES spectra (no. 1–4) under varying voltages *vs.* the Ni/NiO equilibrium (*U*_WE–Ni/NiO_).

**Fig. 7:**
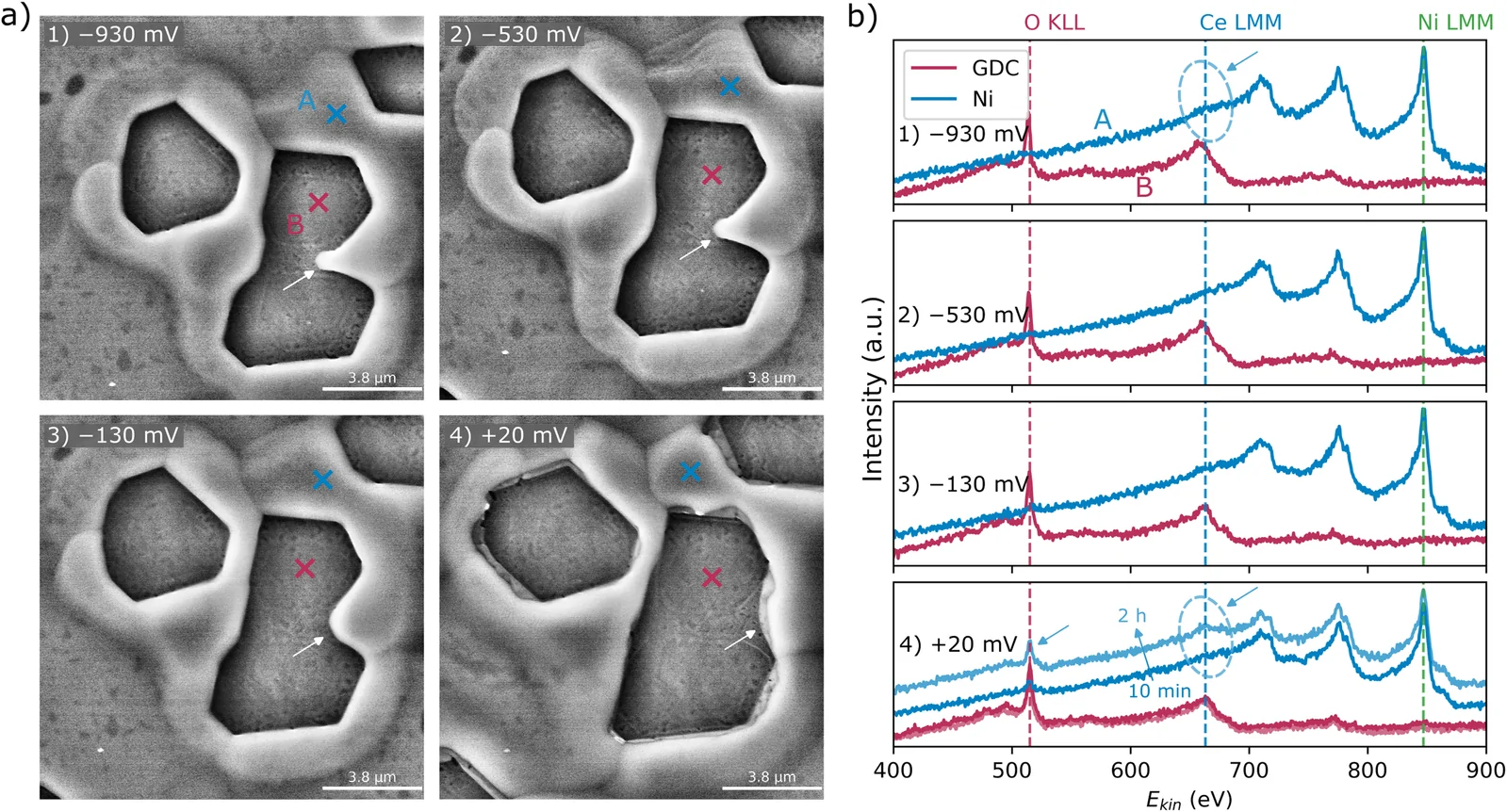
(a) SEM images with markings indicating the locations A and B where the spectra were recorded. (b) AMES spectra under different *U*_WE–Ni/NiO_ (no. 1–4). Both, the SEM in (a) and AMES in (b) were conducted at 750 °C in UHV.

Taking a look at the EXACT-SEM images in Fig. 7a, we observe minor changes in the wetting behaviour of Ni on GDC within the measurement timeframe under reducing conditions (no. 1–3). Only the sharp Ni tail extending into the middle of the image (white arrow) is slowly retracted within the course of the measurement, but this happens independent of the applied cathodic voltage. However, under oxidising conditions (no. 4), more severe changes occur, and the additional NiO phase appears at the TPB.

Analysing the corresponding AMES spectra in Fig. 7b, we observe that no large changes in neither the Ni (region A) nor the GDC (region B) spectra were observed for none of the cathodic conditions (no. 1–3). Only under highly reducing conditions at −930 mV, a very subtle shoulder at 663 eV (Ce LMM) may become visible in the spectrum measured on the Ni film (no. 1, blue curve), as is indicated by the blue arrow. Under oxidising conditions (+20 mV *vs.* Ni/NiO, spectra no. 4), however, additional peaks at 515 eV (O KLL) and 663 eV (Ce LMM) become visible in the spectra acquired on Ni (region A, blue curve). These changes are especially pronounced in the spectra recorded after 2 hours of measuring under oxidising conditions (lighter blue line in Fig. 7b, no. 4, indicated by blue arrows). While the Ni spectrum changed under oxidising conditions, the GDC spectrum remained practically identical – compare red and lighter red spectrum, which are almost indistinguishable.

After every spectrum recorded in Fig. 7, Auger–Meitner mappings were carried out to spatially correlate chemical information. The resulting mappings for O KLL (515 eV), Ce LMM (663 eV) and Ni LMM (847 eV) are depicted in Fig. 8 as red, blue, and green intensity maps, respectively. The column to the right labelled ‘RGB overlay’ shows the normalized sum of the intensity and thus the RGB values from the individual elements.

**Fig. 8:**
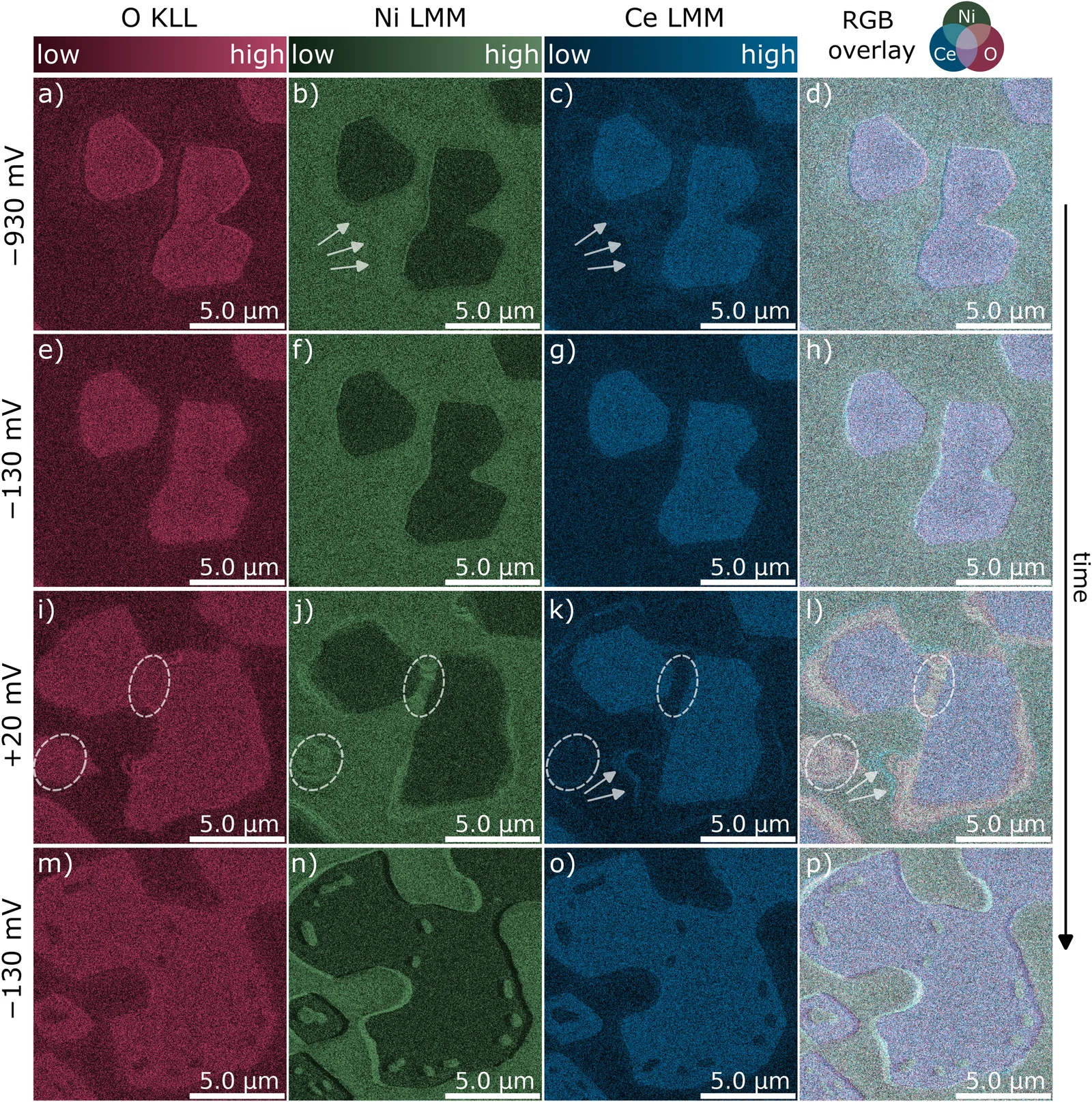
EXACT-AMEM mappings under varying voltages *U*_WE *vs.* Ni/NiO_ at 750 °C for Ni LMM, O KLL, Ce LMM ((a)–(c), (e)–(g), (i)–(k) and (m)–(o)) and the RGB overlay of the indivudal mapping signals ((d), (h), (l) and (p)). The intensity scale of each individual mapping is normalized.

Under reducing conditions (Fig. 8a–h), rather distinct phases are visible: one characterized by a high Ni signal accompanied by low O and Ce signals, and the other by high O and Ce signals accompanied by a low Ni signal (please note that small overlaps in Ni and O signals stem from a time effect since the O KLL mappings were taken after the Ni LMM mappings. Within this timeframe, the Ni film slowly undergoes dewetting, leading to an apparent correlation between Ni and O, which is thus an artifact and does not indicate NiO formation). We therefore conclude that under conditions with *U*_WE–Ni/NiO_ < 0 mV Ni and GDC are clearly separated from each other, where Ni is definitely present as metal, as there is no spatial correlation between Ni and O (Fig. 6a–h).

Interestingly, under strongly reducing conditions at −930 mV *vs.* Ni/NiO, small amounts of Ce and O are observed also on top of the Ni layer in a region surrounding the pinholes in the Ni thin-film, as indicated by the white arrows in Fig. 8b and c and the blue arrow in Fig. 7b (no. 1). This effect is rather subtle but especially in the Auger–Meitner mappings clearly visible by the Ce and O halo around the TPB (see also the RGB overlay in Fig. 8d). Potentially two different mechanisms may explain this appearance of Ce on the Ni film under such strong cathodic polarization: first, as Ce–Ni intermetallic phases can form under highly reducing conditions,^79^ Ce may be partially reduced to a metallic state at the GDC/Ni interface, dissolve into the Ni lattice, and subsequently segregate to the Ni surface. Second, the strongly reduced state of GDC (possibly a bixbyite-type structure^67^) may modify CeO_*x*_–Ni interactions,^80^ promoting cerium oxide enrichment or overgrowth at the Ni surface. Since the halo around the TPB exhibits both Ce and O intensity and both signals vanish as soon as the conditions become rather mildly reducing (−130 mV, Fig. 8f and g), the second explanation appears to be the more likely scenario.

Upon oxidation, the sharply defined boundaries between the distinct phases start to blur – see Fig. 8i–l. Areas that were previously clearly assigned to Ni close to its boundary with GDC now exhibit high Ni as well as high O signal intensities while the Ce signal stays low, as indicated by dashed ellipses in Fig. 8i–l. This is most clearly evident in the RGB overlay in Fig. 8l, where the overlapping high intensities of Ni (green) and oxygen (red) become visible as extended yellowish regions.

We therefore conclude that at *U*_WE–Ni/NiO_ > 0 mV the *in situ* formation of NiO can be clearly confirmed spectroscopically (Fig. 8i–l), whereby the observed Ni oxidation onset voltage coincides perfectly with thermodynamic data. Interestingly, the Ni layer is not fully oxidized, but a large fraction of the film remains metallic. Apparently, as already mentioned in the discussion of the EXACT-SEM results above, this oxidation initially takes place at the Ni/GDC interface and slowly proceeds towards the Ni surface *via* the oxidized TPB and the Ni atop the NiO retracts from the TPB *via* accelerated dewetting. As discussed above, the NiO layer thickness estimated from the coulometric titration data (Fig. 4a) is approximately 8 nm. This value qualitatively agrees with our EXACT-AMEM results, since the NiO front does not penetrate the entirety of the film, and is also in line with literature reports on the relatively high resistance of Ni against oxidation.^73,74,76,77^

Furthermore, upon oxidation, by looking at the Ce LMM region in Fig. 8k, it is noticeable that Ce intensity also appears in areas close to the Ni/NiO interface. This can be observed as a thin band that surrounds the pinholes in the Ni film at slight distance – it is marked in Fig. 8k with two white arrows at the point of its highest Ce intensity. In the RGB overlay, this feature is nicely visible as a turquoise rim adjacent to the yellowish NiO regions. The Ce LLM region was measured after the Ni LMM and O KLL regions, introducing a time delay during which additional Ni oxidation may have occurred. This possible time artifact prevents a clear distinction between Ce residing on metallic Ni or on NiO.

We therefore interpret the enhanced Ce signal observed on Ni or NiO regions as evidence for ceria migration under conditions leading to nickel oxidation. This migration could involve ceria wetting either metallic Ni or oxidized NiO, or even the formation of a distinct phase, such as CeNiO_3_ or Ce_1−*x*_Ni_*x*_O_2_.^81,82^ Interestingly, morphological evolution of GDC has been observed in both porous^20^ and thin-film^21^ Ni/GDC electrodes. These studies report ceria redistribution, which are strikingly similar to our observations, primarily under high humidity (*i.e.* less reducing) conditions. While our data does not allow for definitive conclusions, several plausible scenarios may explain the observed redistribution of cerium under anodic polarization:

One possible explanation could be that the NiO formation alters the metal/oxide–support interactions, which in turn could enhance surface mobility of ceria. This hypothesis aligns with observations in Fig. 6d ande, where enhanced diffusivity of NiO on GDC was discovered under oxidation conditions. Similarly, wetting/overgrowth phenomena of ceria on forming NiO during reoxidation might be favoured as oxide–oxide interfaces generally allow more compatible oxygen-mediated bonding and lower interface energies than oxide–metal interfaces.^83^ Enhanced ceria mobility could also result from polarization-induced changes in interfacial energies as Ni–ceria interactions may strongly be coupled to Ce oxidation states.^84,85^

Another mechanism could involve the dissolution of metallic cerium into the Ni lattice under strongly reducing conditions, forming Ce–Ni intermetallic phases. Upon oxidation, this dissolved Ce may migrate to the Ni surface and re-oxidize into cerium oxide. This explanation would account for the accumulation of Ce on Ni during anodic polarization (however, for the abovementioned Ce overgrowth on Ni under strongly cathodic polarization in UHV, where no external oxygen source is available, this mechanism becomes less plausible).

Despite these plausible explanations, the exact driving forces behind cerium redistribution remain unresolved. However, given the complexity of the dynamically evolving Ni/GDC interface under strong redox polarization, a rigorous mechanistic clarification would require dedicated studies with complementary techniques and extensive theoretical treatment beyond the scope of the present work. Nevertheless, the direct observation of reversible cerium redistribution itself represents an important finding, as it highlights the highly dynamic nature of the Ni/GDC interface under electrochemically relevant conditions and may serve as an experimental basis for future computational studies on the underlying interfacial energetics and redox-driven interface evolution.

Finally, after the oxidation step, another reduction at *U*_WE–Ni/NiO_ = −130 mV was carried out (Fig. 8m–p). Both effects, the spatial correlation between Ni and O, and the high Ce signal on the Ni metal, which appeared under oxidising conditions, vanish and the distinct boundaries between Ni and GDC are again visible. Furthermore, substantial Ni-dewetting and the formation of isolated Ni droplets is observed.

Taken together, these observations show that Ni and GDC remain clearly phase-separated under reducing conditions, with only trace Ce enrichment on the Ni surface under strongly reducing conditions. Upon oxidation above the Ni/NiO threshold, a new NiO phase forms near the TPB, accompanied by Ce enrichment at the Ni/NiO/vacuum triple phase boundary. Re-reduction after anodic polarization leads to rapid dewetting and pronounced morphological change.

### 3.2 *In situ* imaging of Ni/GDC dynamics on 3D porous electrodes in vacuum

Having established these morphological and surface-chemical effects on Ni/GDC thin-film model electrodes, we next examine whether they translate to more realistic SOC electrode geometries. This chapter presents analogous UHV experiments on 3D porous samples, testing in particular whether redox-cycling-induced Ni-dewetting, established on thin-films, persists in more complex electrode structures.

As such ‘real’ structures – meaning electrodes that are used in industrially fabricated SOFCs/SOECs, which also show morphological similarities with metal/oxide composite catalysts – are obviously optimized towards high performance, they require high surface areas. Therefore, they do not resemble thin-films but exhibit 3D porous structures, consisting of the interpenetrating, percolating phases Ni, GDC, and gas. We therefore investigate a 3D porous Ni/GDC electrode structure that more closely resembles a real SOFC/SOEC fuel electrode, while remaining in UHV conditions using the EXACT technique with oxygen buffering CE.^39^ The aim here is to gain further understanding about the difference in morphological and surface-chemical behaviour of thin-films *versus* application-oriented 3D porous electrodes.

Considering the transmission line concept discussed in Section 2.2.1, the absence of a DC current in UHV implies that the oxygen stoichiometry and effective *p*O_2_ of the entire porous electrode changes homogeneously with the cell voltage. However, as we have already seen on the model-type Ni/GDC electrodes, when we apply voltages under which we start oxidising the Ni particles (*U*_WE–Ni/NiO_ ≥ 0), only those parts of the Ni phase in close vicinity to GDC are expected to be oxidised.

#### 3.2.1 Time resolved SEM imaging on 3D porous Ni/GDC electrodes

Time-resolved EXACT-SEM images of a cleaved porous Ni/GDC electrode cross-section (45° observation angle) at 750 °C under varying voltages *vs.* Ni/NiO are depicted in Fig. 9 including the used voltage program (top graph). Larger particles correspond to Ni metal, while the smaller particles are GDC (confirmed with AMEM, see next chapter). Fig. 9a'–c' show magnifications. Like in case of the model-type thin-film samples, to gain a better intuitive view of the morphological dynamics occurring under polarization, we would like to highly encourage the reader to view the provided time-resolved SEM videos in the SI (03_type3_staircase1.mp4, 04_type3_staircase2.mp4).

**Fig. 9:**
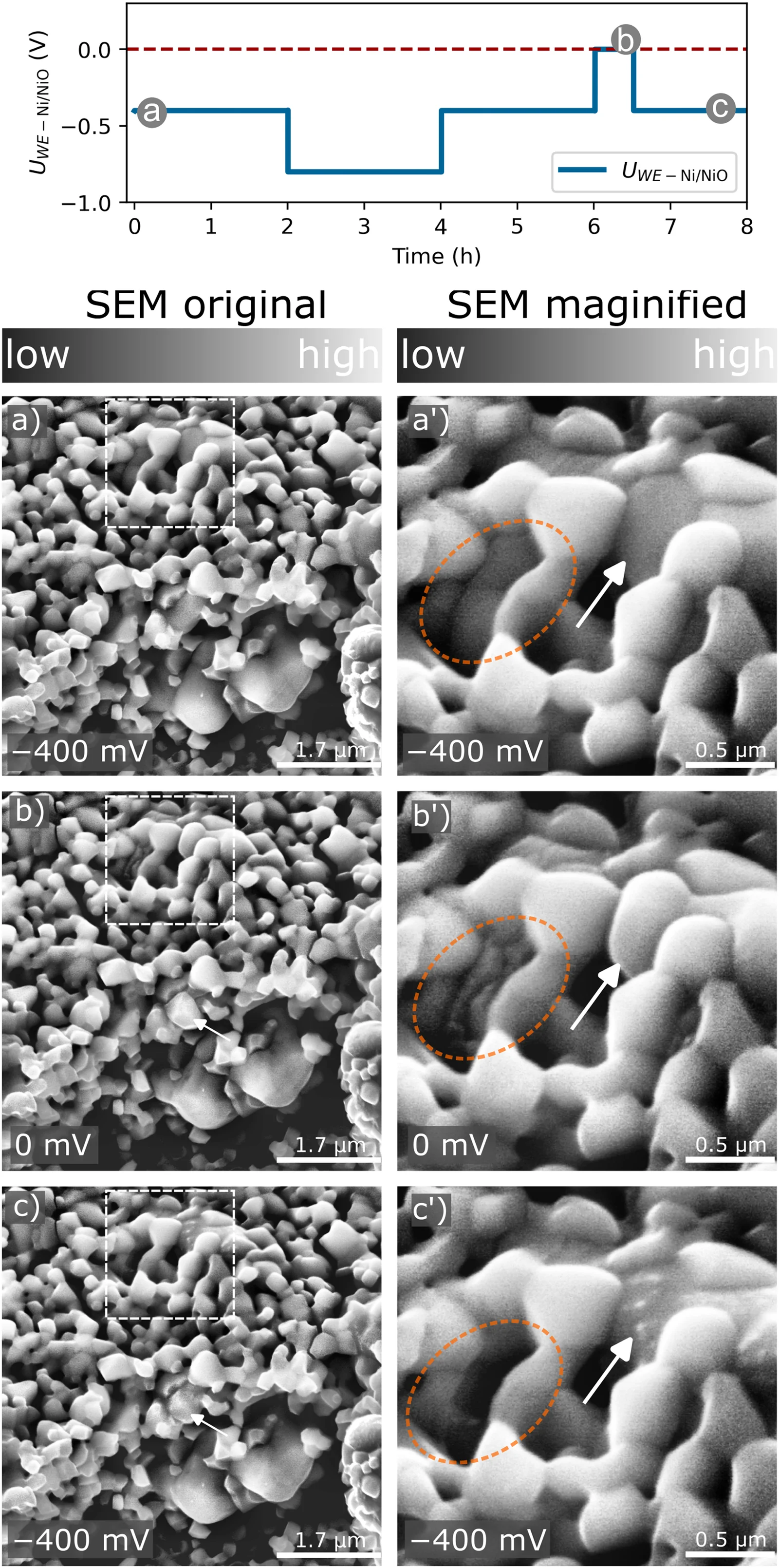
(a–c) EXACT SEM images of a cleaved cross-section of a porous Ni/GDC electrode at 750 °C upon different voltages *U*_WE–Ni/NiO_ in UHV. Grey markers in the top graph indicate the voltage/time coordinates for SEM images. (a'–c') Depict magnified images where the orange ellipses indicate regions of interest for redox-driven Ni re-distribution.

Upon changing the applied voltage from moderate (*U*_WE–Ni/NiO_ = −400 mV) to strongly (−800 mV) reducing conditions, the porous electrode undergoes a breathing-like motion. This becomes evident by looking at the small cracks to the right of the image (03_type3_staircase1.mp4), which are already present at the beginning of the experiment in Fig. 9a. By changing the voltage to more reducing conditions, this gap closes almost completely. This effect is reversed as soon as the voltage is set back to the initial value of −400 mV (03_type3_staircase1.mp4). Other than that, no severe morphological changes are observed while staying below the Ni/NiO threshold.

The breathing-like motion is most likely a consequence of the chemical expansion of GDC upon reduction.^70,71^ This is extremely well observable in the time-resolved SEM video in the SI (05_type3_CV_post_staircase.mp4), where we demonstrate the dynamic changes of the porous electrode at the exact same spot during a multi-cycle CV measurement, which was taken after the measurements shown in Fig. 9. Even though this chemical expansion was to be expected in a way, as it is well-known from literature that doped ceria expands in the low % range when the equivalent *p*O_2_ is reduced,^70,71^ the video mentioned above is particularly eye-opening, as it impressively demonstrates the extent and impact on an application-relevant, porous Ni/GDC structure.

Based on this explanation, the presence of the crack already at the beginning of the experiment is most likely also related to chemical expansion effects. As the porous Ni/GDC WE was sintered in dry hydrogen (5% H_2_ in Ar), it initially experienced strongly reducing conditions, resulting in an expanded GDC lattice. When heating it up under more mildly reducing conditions (*U*_WE–Ni/NiO_ = −400 mV) inside the AMEM, partial electrochemical re-oxidation of GDC lead to lattice contraction and, consequently, crack formation. Upon electrochemical reduction during cathodic polarization, this contraction is reversed and therefore the crack closes.

With regard to Ni oxidation, it is worth noting that this occurs in a slightly different manner on the 3D porous samples than in the thin-film experiments, which can be related to the different sample architecture: instead of having Ni particles on a GDC surface, the WE consists of small GDC particles sitting on larger Ni particles.

In some areas, highlighted by the white arrow in Fig. 9a'–c', right at the Ni/NiO equilibrium at *U*_WE–Ni/NiO_ = 0 mV during oxidation, the Ni metal particles increase in apparent size. This observation gives the impression that the metallic Ni is somehow squeezed into the empty space of the pores, which is why we call this phenomenon “squeeze-the-balloon” effect (see Fig. 13c). This designation implies the assumption that upon oxidation, the NiO formation at the interface with GDC causes expansion, which exerts strain on the Ni/GDC framework. Due to its soft and ductile nature at elevated temperature, metallic Ni can accommodate the resulting strain by undergoing the observed morphological deformation, similar to a ballon, which is compressed irregularly and deflects into the available space due to deformation. More details on this “squeeze-the-balloon” effect will follow in the next subchapter and are illustrated in Fig. 13c.

Furthermore, in the area highlighted by the orange ellipse in Fig. 9a'–c', Ni metal also becomes mobile upon oxidation in a way that was already observed on the thin-film samples. This means, it seems to uncover the newly formed NiO at the Ni/GDC interface, in line with the previous interpretation that Ni on NiO appears to be significantly more mobile than Ni on GDC and, therefore, dewetting proceeds faster upon oxidation than under reducing conditions.

Upon the subsequent re-reduction (*i.e.* changing the voltage back to *U*_WE–Ni/NiO_ = −400 mV), a pore forms at the site where Ni metal was originally present (compare Fig. 9a' and c', orange ellipse). Moreover, also the “squeeze-the-balloon” effect is reversed as soon as the voltage is retracted back to −400 mV. Additionally, small, isolated particles remain on top of the metallic Ni (see Fig. 9c', white arrow). The composition and origin of these particles remain unknown for this sample as the spatial resolution for AMEM mappings was not sufficient (see following chapter). The identification of such particles will be in focus of future works.

In addition to the first redox-cycling step, a second one was carried out for Auger–Meitner analysis. SEM imaging for this second redox-step is provided in the SI Video (04_type3_staircase2.mp4). Upon the second oxidation, the large Ni particles close to the cleaved cross-section at the bottom of the graphs are only partially oxidized as the majority of the particle's surfaces remain metallic within the timeframe of the experiment. One may assume that the Ni particles with high coverage of percolating GDC grains are faster oxidized as diffusion distances are small.

The changes in surface chemistry of our 3D porous Ni/GDC electrodes upon redox-cycling will be discussed in the upcoming subchapter, which includes the AMEM mappings.

#### 3.2.2 *In situ* AMEM of 3D porous Ni/GDC electrodes

Fig. 10 shows AMEM mappings (only the RGB overlays) of the cleaved cross section obtained at the exact same location as the SEM images in Fig. 9. On the left, SEM images at the corresponding conditions are depicted. Fig. 10a and a' display images of the pristine sample after heating up at *U*_WE–Ni/NiO_ = −400 mV. Under these reducing conditions, rather distinct and clearly separated Ni and GDC phases are visible in the Auger–Meitner mappings and no spatial correlation between Ni and O is observed.

**Fig. 10:**
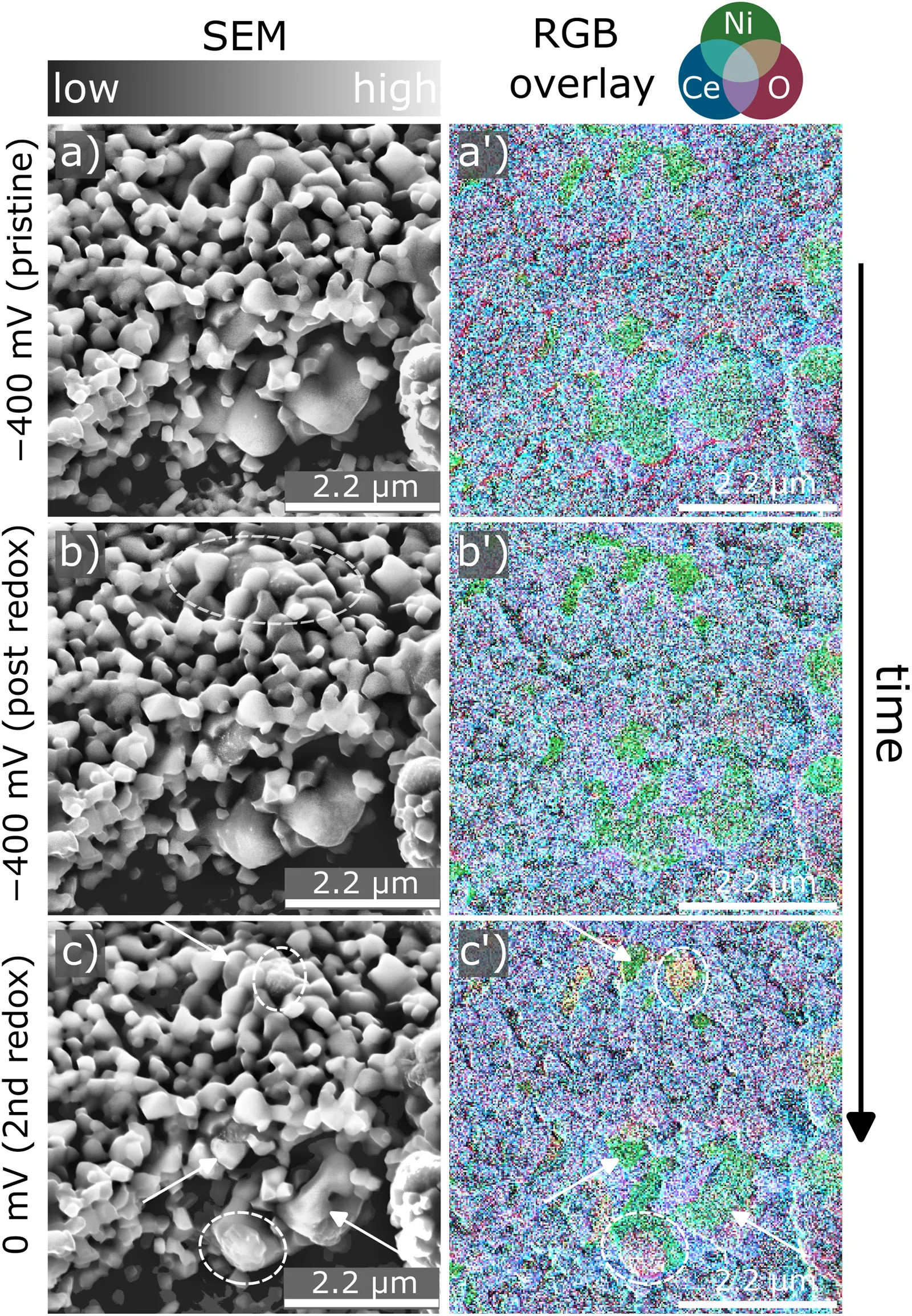
SEM images (left column) and EXACT-AMEM mappings (right column) of a cleaved cross-section from a 3D porous Ni/GDC electrode at 750 °C upon varying voltages *U*_WE–Ni/NiO_ in UHV under the following conditions: pristine at −400 mV after heating up (a and a′), after the first redox-cycling at −400 mV (b and b′), and upon the second oxidation (c and c′).

SEM and AMEM images of the sample under reducing conditions after the first redox-cycling step from the previous chapter are displayed in Fig. 10b and b'. From AMEM mappings, we cannot identify the small particles visible in the SEM image (white ellipse), which appear after the first re-reduction as besides high Ni signals, no additional O or Ce species seems to be present.

Upon the second oxidation, strong changes in morphology and surface chemistry are again visible. We highly recommend watching the time-resolved SEM video for this second oxidation, which is provided in the SI (04_type3_staircase2.mp4). The newly formed rougher phases clearly correspond to nickel oxide, as shown by the AMEM mappings (white ellipses in Fig. 10c'), where the NiO is nicely visible as yellowish regions. However, in the video 04_type3_staircase2.mp4, there are also some areas where a much smoother and more roundish shaped phase becomes mobile. These spots are marked by white arrows in Fig. 10c and c'. This phase apparently corresponds to metallic Ni as neither the O KLL, nor the Ce LMM signals are high. We therefore conclude that similar to our results from thin-films, where metallic Ni becomes much more mobile when it sits on NiO, a large proportion of the Ni particles remain metallic and their mobility is enhanced upon redox-cycling.

To further elucidate the so far unidentified particles on Ni that appear after redox-cycling, an additional measurement with higher resolution was performed analogous to the one described above (also on a cleaved cross section of the same sample type). Compared to the previous experiments, the potential of the counter electrode (and thus the effective *p*O_2_ at the CE) was less reliable, resulting in higher uncertainty in *U*_WE–Ni/NiO_. Nevertheless, the distinction between reducing and oxidising conditions remained unambiguous. The large difference in effective *p*O_2_ at the WE between the reducing and oxidising regimes allows for a clear qualitative interpretation of the observed changes, rendering the results robust despite the increased voltage uncertainty. Hence, we denote the applied conditions as “reduction” and “oxidation”. The obtained EXACT-SEM images and AMEM mappings are depicted in Fig. 11.

**Fig. 11:**
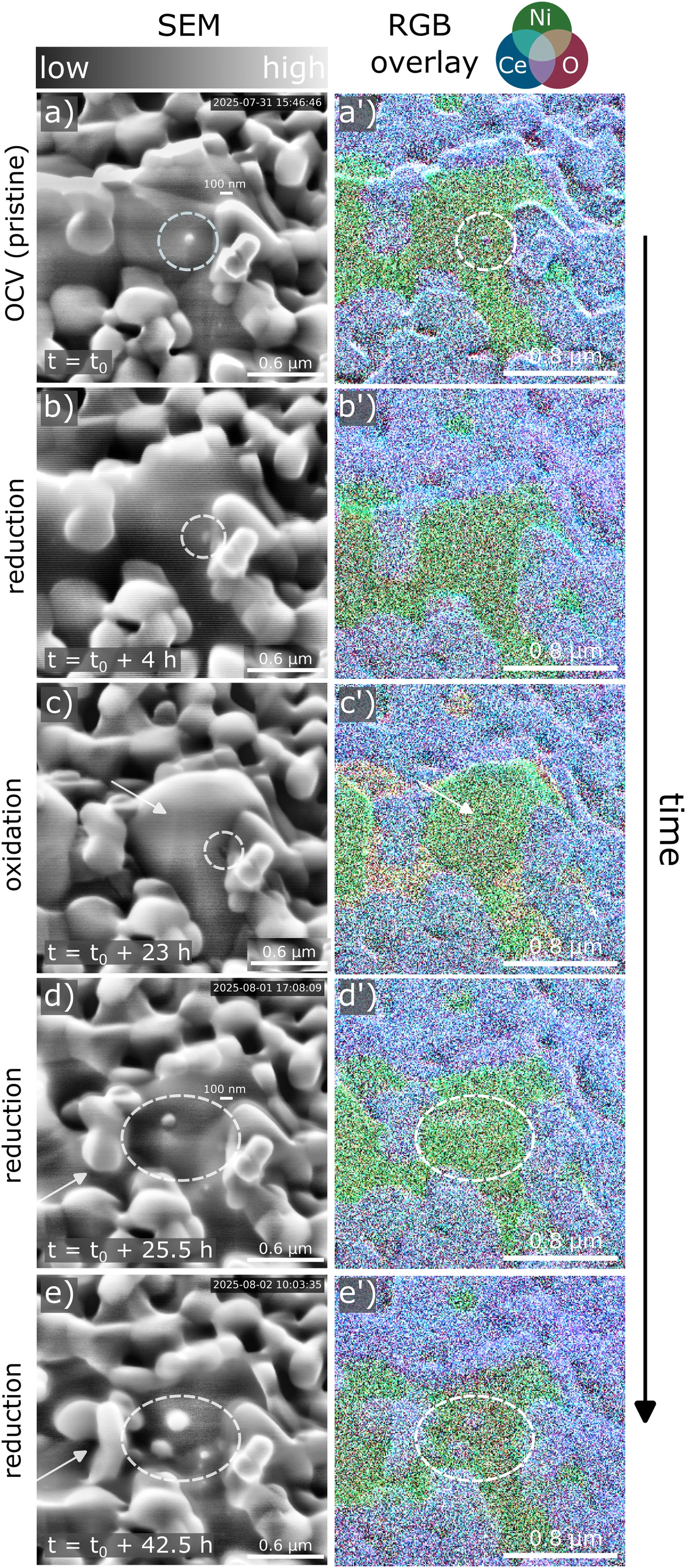
EXACT-AMEM mappings including SEM images of a second cleaved cross-section of a porous Ni/GDC electrode at 750 °C upon different conditions in UHV: the pristine sample at OCV after heating up (a and a'), the sample under reducing conditions (b ans b'), under oxidising conditions (c and c'), and the sample upon the re-reduction (d, d', e and e').

The pristine sample after heating up already showed a tiny particle with a diameter of roughly 80 nm sitting on the Ni surface (white circle in Fig. 11a andb), which corresponds to a ceria particle, as Ce and O signals are high while the Ni signal is low. Notably, we do not have any information about the presence or absence of Gd, as its signal is too low for AMEM detection. After some time and a voltage change to more reducing conditions, this particle starts moving towards the GDC to the right of the Ni particle (white circle in Fig. 11a and b). This moving GDC particle on Ni is well visualized in the SI Video 06_type3_staircase1.mp4.

Upon oxidising voltage (Fig. 11c'), NiO appears in some regions close to the GDC particles (see yellowish regions in the RGB-overlay). This behaviour that nickel is only partially oxidized upon applying anodic voltages, is in line with the observations on both the model-type thin-film electrodes and the first 3D porous Ni/GDC electrode.

Furthermore, the central part of the Ni particle (white arrow in c and c') increases in apparent volume, which may again be explained by the “squeeze-the-balloon” effect (see SI Video 07_type3_oxidation.mp4). Here, with the higher resolution of the AMEM measurements, it can be seen quite clearly that this “inflated” part does indeed correspond to metallic Ni rather than nickel oxide, as no significant increase in the O KLL signal is visible in the AMEM mapping. When the sample is re-reduced (Fig. 11d and 08_type3_reduction.mp4), the “squeeze-the-balloon” effect again disappears quasi-reversible as the Ni particle changes in shape and loses apparent volume.

After some time of re-reduction, small GDC particles appear on the Ni surface as the morphology of Ni alters over time (see 09_type3_GDC_nanoparticle_formation.mp4). This effect may be related with the multiscale GDC mobility reported by Sciazko *et al.* for Ni/GDC anodes operated in humidified hydrogen.^20^ Their work demonstrated that the strong metal–support interaction (SMSI) between Ni and GDC drives GDC redistribution over microns distances, resulting in thin layers of GDC on Ni, which leads to pronounced local changes in triple-phase boundary structure and activity. Our observations suggest that similar dynamic rearrangements may also occur under electrochemical polarization at the nanoscale. The underlying mechanisms, however, remain insufficiently understood, particularly regarding the driving forces for ceria mobility and its dependence on local redox state, overpotential, and surface termination. Moreover, whether this GDC particle formation on Ni in case of 3D porous electrodes is related to the ceria overgrowth effect on Ni, which was observed on the thin-film samples, cannot be unambiguously concluded from the data presently available. These findings, nevertheless, highlight that the Ni/ceria system exhibits a far more complex and dynamic behaviour than commonly assumed. A deeper mechanistic understanding of these coupled nanoscale and microscale processes – and their impact on electrochemical performance – will be an important direction for future work.

### 3.3 Summary of Ni/GDC dynamics in UHV

In summary, EXACT-SEM and AMEM measurements revealed that the 3D porous electrodes display all the effects observed in the thin-film model samples under UHV conditions, along with some additional phenomena specific to an application-relevant 3D porous system. Fig. 13 sketches an overview of our observations for thin-films and 3D porous cermets.

On both thin-film and porous electrodes, the absence of pronounced Ni dewetting under strongly reducing conditions in Ni/GDC stands in marked contrast to degradation models commonly discussed for state-of-the-art Ni/YSZ composite electrodes. This finding contrasts the common assumption that high cathodic polarization is the main driver of dewetting effects in metal/ceramic composite electrodes. Instead, our results demonstrate that crossing the metal oxidation threshold constitutes the decisive trigger for accelerated morphology changes in Ni/GDC, highlighting that redox effects have been strongly underestimated in previous interpretations.

Our EXACT-AMEM results clearly show that the presence of NiO drastically increases the mobility of Ni. In case of thin-films, this is evident from greatly accelerated dewetting of the Ni film. For 3D porous frameworks, this leads to an increase in porosity in certain areas. Upon re-reduction, small, possibly ceria-based particles remain on the Ni surface.

Such GDC migration was observed for both types of samples. With respect to thin-films, GDC migration on Ni metal was observed under strongly reducing and oxidising conditions. Under strongly reducing conditions, a subtle halo of increased Ce-intensity was observed next to the TPB. Under oxidising conditions, Ce/Ce-oxide enrichment was found in the vicinity of the Ni/NiO/vacuum triple phase boundary. In 3D porous samples, by transitioning from high towards lower *p*O_2_ conditions, small ceria nanoparticles were formed on Ni surfaces. A connection between the Ce signal observable on Ni under certain conditions in the model system and the ceria mobility on Ni in the real system seems evident but cannot yet be conclusively proven.

These findings suggest that redox-cycling should not only be regarded as detrimental but can be deliberately employed as a design parameter to tailor electrode morphology. Such a perspective represents a conceptual shift from degradation avoidance towards intentional morphology engineering *via* controlled redox treatments. In fact, redox-cycling is already implicitly utilized in the present work to generate defined porosity in our thin-film samples (see Section 3.1.2), underscoring its potential as a powerful tool for microstructural optimization.

Finally, on 3D porous electrodes, we discovered a phenomenon, which to the best of our knowledge was not observed so far, and which we described as “squeeze-the-balloon” effect. A significant change in Ni morphology was observed when switching to oxidising *p*O_2_ conditions above the Ni/NiO transition, manifested as an apparent volume increase of metallic Ni particles. As sketched in, this can be explained by the volume increase of our NiO interface oxide which causes strain to the Ni/GDC framework. Since the GDC framework is rigid, the imposed strain is primarily accommodated by the soft and ductile Ni metal particles, which adjust their shape accordingly.

With regard to the chosen novel combination of methods – EXACT-AMEM – it should be emphasised here that the ability to directly correlate local redox state, surface chemistry, and morphology in real time highlights the broader potential of our approach. Rather than being limited to the present Ni/GDC system, this methodology is applicable to a wide range of mixed conducting oxides and oxide-supported metal catalysts, where dynamic changes at metal–oxide interfaces govern performance and stability. By decoupling oxygen chemical potential from gas-phase chemistry, EXACT-AMEM provides a unique experimental platform for *in situ* investigations of dynamic interfaces under well-defined thermodynamic driving force. To validate whether these effects persist in the presence of a gas phase, we conducted near-ambient pressure XPS measurements under conditions mimicking SOFC/SOEC operation, as presented in the following section.

### 3.4 Effect of gases on Ni/GDC dynamics studied by near ambient pressure X-ray photoelectron spectroscopy

In the previous chapter, we have demonstrated the effect of polarising our electrodes at high temperatures using the EXACT technique under UHV conditions. These measurements provide direct access to polarization-induced morphological changes, but the UHV environment does not include gas-phase species present during technologically relevant operation. Therefore, in the current chapter, these findings are further examined under similar effective *p*O_2_ conditions, but with the inclusion of gases and electrochemical polarization during NAP-XPS measurements to assess whether the same qualitative trends are also observed in a gas-phase environment under the specific near-ambient-pressure conditions used here. Considering the effect of gases is on the one hand important as they are relevant for high-temperature electrolysis, and on the other hand because adsorbates originating from the gas phase can substantially alter the surface energies of the materials and thus affect morphological changes of Ni/GDC electrodes.

The EXACT-SEM results above show that dewetting of Ni/GDC under reducing conditions proceeds very slowly and is substantially accelerated by about an order of magnitude in the presence of a NiO interface during redox-cycling. To test whether these findings remain true in the presence of a gas phase, near ambient pressure X-ray photoelectron spectroscopy (NAP-XPS) was carried out on model-type thin-film samples (type 1). Here, polarising the WE at high temperatures is possible under gas atmospheres with total pressures in the order of 1 mbar.

Results from NAP-XPS (obtained on electrode type 1) are depicted in Fig. 12. Fig. 12a shows the XPS spectra of the Ce 3d and Ni 2p regions. As the two regions can be separated using a well-established fingerprint fitting,^39^ the projected Ni coverage *θ*_Ni,XPS_ can be monitored using the Ni/Ce XPS intensity ratios including several correction factors according to eqn (12) and (13). Therein, AR_Ni/Ce_ corresponds to the area ratio of the two phases, Ni and GDC. *I*_i_, *σ*_i_, IMFP_i_, and *ρ*_i_ correspond to the transition specific XPS intensity, the XPS cross-section, the inelastic mean free path and the elemental density of the species i, respectively. It should be noted that *θ*_Ni,XPS_ serves as a semi-quantitative, XPS-derived coverage descriptor suitable for comparing temporal trends within this experiment, rather than as a kinetic metric with universal applicability at device level. Together with the applied voltage (again given as *U*_WE–Ni/NiO_), the projected Ni coverage is plotted against time in Fig. 12b. The vertical dashed grey line indicates a switch in gas composition from a H_2_/H_2_O = 1 (*p*_tot_ = 0.4 mbar) to a CO/CO_2_ = 0.8 (*p*_tot_ = 0.5 mbar) atmosphere. What should be emphasized here is that for both gas compositions, the H_2_/H_2_O and CO/CO_2_ ratios are adjusted in such a way that both correspond to the same equivalent *p*O_2_ of 3·10^−20^ bar at 750 °C, which corresponds to a voltage of about –230 mV *vs.* Ni/NiO.^64^12
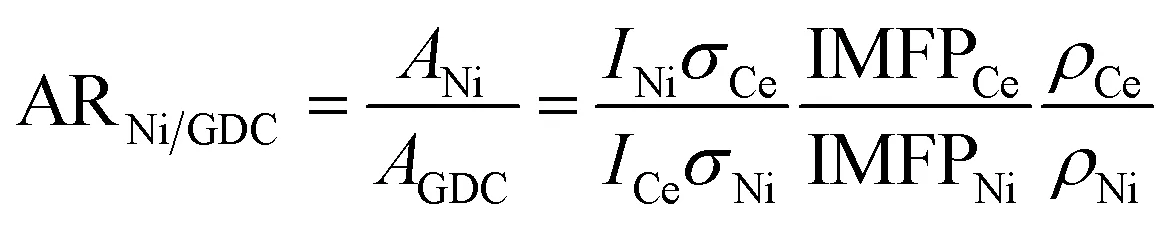
13
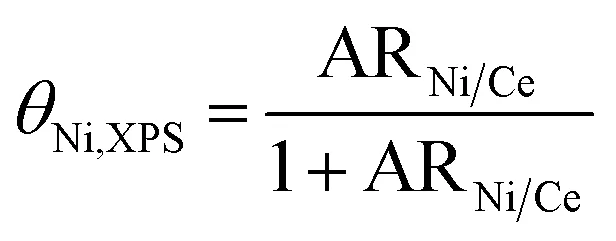


**Fig. 12:**
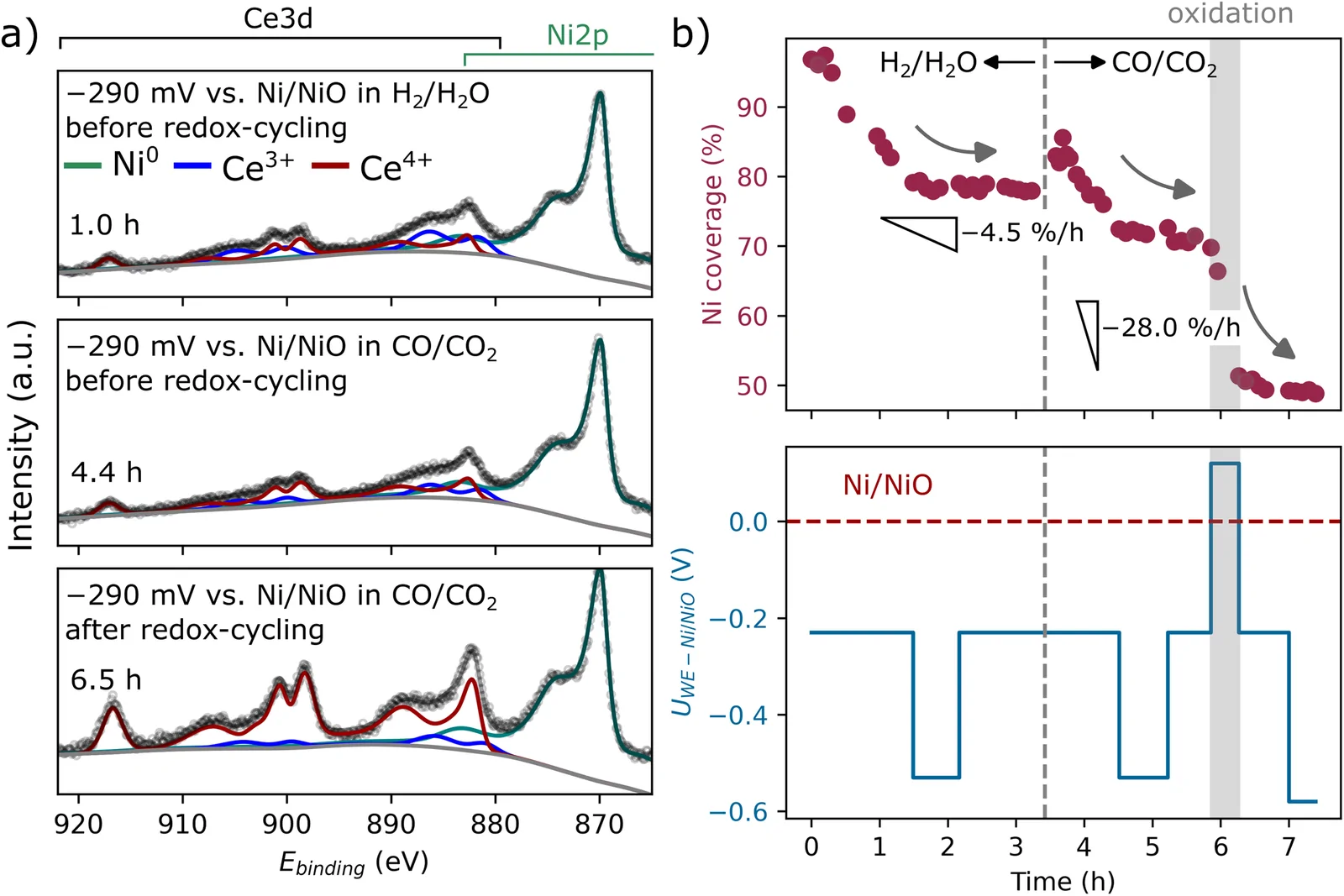
NAP-XPS results for Ni-dewetting on GDC at 750 °C under varying voltages *vs.* Ni/NiO: (a) XPS spectra at −290 mV *vs.* Ni/NiO for the overlapping Ni 2p and Ce 3d region under H_2_/H_2_O and CO/CO_2_ atmospheres before and after redox-cycling. (b) Evolution of the Ni/Ce XPS fraction and the applied voltage *vs.* Ni/NiO against time. The vertical grey dashed line in (b) indicates the gas switch from a H_2_/H_2_O to a CO/CO_2_ atmosphere.

At the start of the experiment, almost no Ce 3d signal intensity is visible under H_2_/H_2_O conditions at 750 °C. Without changing the voltage, the Ni/Ce fraction slowly decreases over time until it reaches a stable value after about 1.5 h, as indicated by the first grey arrow in Fig. 12b. Even, when the voltage changes to more cathodic values, the Ni/Ce fraction remains unaffected.

Upon the change in gas composition from H_2_/H_2_O to CO/CO_2_ after roughly 3.5 h, the Ni/Ce fraction seems to be altered slightly. Once the new gas phase is established, however, a constant Ni/Ce fraction is reached again after roughly 5 h (*ca.* 1 h after the gas exchange). This effect of the gas exchange on the Ni/Ce fraction indicates that changes in adsorbate chemistry do indeed have a subtle influence on the wetting behaviour of Ni and GDC, albeit not a particularly significant one. Also, in CO/CO_2_, a more cathodic polarization did not significantly affect the wetting behaviour. However, when exceeding the Ni/NiO threshold by applying a sufficiently high anodic voltage (after roughly 6 h; see grey bar in Fig. 12b), the Ni/Ce fraction decreases quickly even within a relatively short timeframe of the oxidising treatment. Also, after the subsequent reduction, the amount of observed Ce in XPS is further increased slowly.

For reducing and redox-cycling conditions, the calculated dewetting rates, defined here as the slope of the projected Ni coverage *θ*_Ni,XPS_*versus* time (expressed in % Ni coverage per hour) are −4.5%/h and −28.0%/h, respectively. As for the EXACT-AMEM experiments (see Section 3.1.2), these values are not intended as universally valid kinetic metrics, but as experiment-specific descriptors for semi-quantitatively comparing different polarization/gas segments within our NAP-XPS measurements. Under these specific NAP-XPS conditions, redox-cycling thus accelerates dewetting markedly compared to purely reducing conditions, by an empirical factor of approximately six within this dataset. While this factor is specific to the present operating conditions, it confirms the same qualitative trend observed independently in EXACT-SEM: Ni redistribution is comparatively slow under reducing conditions, but is strongly accelerated once the Ni/NiO threshold is exceeded.

In summary, the NAP-XPS results follow the same trend as observed for Ni-dewetting in UHV in the previous chapter. Changes of the voltage below the Ni/NiO transition threshold do not substantially influence dewetting (at least within the relatively short timeframes of the experiment). The gas atmosphere appears to have small, but clearly observable effects. Redox-cycling, on the other hand, strongly accelerates dewetting by a factor of roughly 6. While these results may seem redundant as they qualitatively mirror previous experiments in UHV in, they are essential for understanding dewetting under more realistic SOFC/SOEC operating conditions. As soon as gases are present, adsorbates on the surfaces of GDC and Ni could strongly alter the wetting behaviour compared to UHV-based EXACT experiments. Quantitatively, this also seems to be the case as the rate of dewetting including gases in our NAP-XPS study is significantly larger compared to our measurements in UHV. A possible reason for the stronger dewetting in SOFC/SOEC-relevant atmospheres is that adsorbates such as hydroxyl or carbonate groups may selectively lower the surface energy of GDC, thereby leading to stronger Ni dewetting.^86^

Furthermore, the acceleration of dewetting using redox-cycling is more pronounced in UHV with an acceleration factor of more than 10 compared to a factor of 6 in case of NAP-XPS. Nevertheless, the clear parallels between the measurements in UHV and the NAP-XPS experiments emphasize the high relevance of the EXACT-AMEM results for a detailed understanding of Ni/GDC and related catalyst-systems.

One main difference between NAP-XPS and UHV experiments is the influence of the gas phase on the formation of NiO. In the NAP-XPS, as we are working with reducing gases, the NiO species grown *in situ* upon anodic polarization is expected to be restricted to the Ni/GDC interface and is likely not stable in contact with the gas phase at the TPB. This can be verified as we did not observe a NiO species in the Ni 2p region. Similar results were already found for exsolved Ni nanoparticles on perovskite-type oxides, as Ni cannot be fully oxidized when the atmosphere counter-acts as reducing driving force from the surface.^87^ Therefore, we can conclude that enhanced Ni-dewetting upon redox-cycling remains an important factor under high-temperature electrolysis conditions.

## 4 Conclusions

In this work, we explored the dynamics of the high temperature (electro-)catalyst system Ni/Gd-doped ceria (GDC) using complementary *in situ* microscopy and spectroscopy techniques. A key methodological advance of this work is the introduction of *in situ* Auger–Meitner electron microscopy (AMEM) to the field of high-temperature electrolysis, enabled by employing the Electrochemical oXygen Activity ConTrol (EXACT) technique. This approach enables direct observation of morphological and surface-chemical dynamics at metal–oxide interfaces under precisely controlled redox conditions.

On the model system Ni/GDC (summarized in Fig. 13), we find that Ni dewetting is minor under purely reducing conditions, where Ni remains metallic, but is strongly accelerated once the Ni/NiO threshold is exceeded during redox-cycling. The same qualitative trend is observed under both UHV and NAP-XPS conditions (H_2_/H_2_O and CO/CO_2_ atmospheres), as well as for both thin-film model electrodes and 3D porous composite electrodes.

**Fig. 13:**
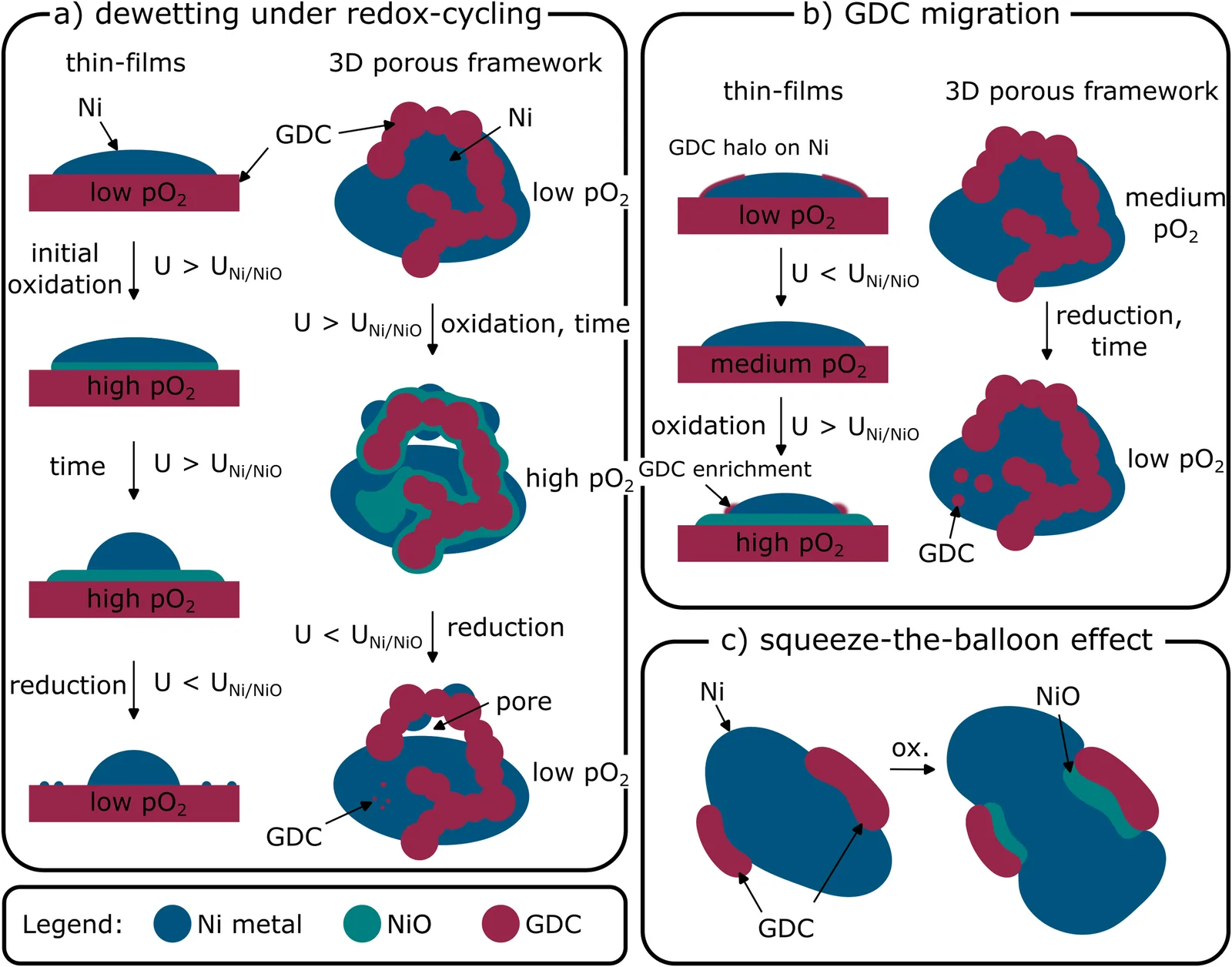
Summary of (a) dewetting under redox-cycling, (b) GDC migration upon changing the equivalent *p*O_2_ in UHV using the EXACT technique for thin-films and 3D porous samples. (c) Sketch of the squeeze-the-balloon effect of Ni/GDC composites upon electrochemical oxidation. As the oxidation is facilitated at the Ni/GDC interface, the nickel oxide at the interface causes expansion which exerts strain on the Ni metal.

In parallel, we observe oxide redistribution processes, including ceria migration onto the Ni surface under both reducing and oxidising conditions on thin-film model electrodes, and GDC nanoparticle formation on Ni metal in 3D porous electrodes upon strong electrochemical reduction. Particularly in 3D porous samples, we further observe reversible Ni particle reshaping upon redox-cycling (described as “squeeze-the-balloon” effect), and pronounced chemical expansion/contraction of the electrode during polarization changes.

Beyond identifying redox-driven dewetting as a degradation mechanism relevant to SOC operation, these results point to controlled redox-cycling as a potential route for microstructure engineering, shifting the perspective from passive degradation management toward active, redox-based design of electrode morphology in energy materials.

## Conflicts of interest

There are no conflicts of interest to declare.

## Supplementary Material

TA-014-D6TA04877C-s001

## Data Availability

Data for this article, including raw impedance data, DC coulometry data and XPS spectra are available at TU Wien Research Data at https://doi.org/10.48436/kh1ts-trj53. Supplementary information (SI): *in situ* SEM videos paired with the voltage programs, details on electrode preparation of 3D porous Ni/GDC composite electrodes, SEM images of thin-film cross-sections, EIS fitting, thin-film XRD and SEM image segmentation. See DOI: https://doi.org/10.1039/d6ta04877c.
