# Supplementary material for: Visualising redox-driven metal–oxide interface dynamics in Ni/GDC electrodes via in situ Auger–Meitner electron microscopy
Source: J Mater Chem A Mater. 2026 Oct 7;14(64):44763–89. doi: 10.1039/d6ta04877c (PMC13644783; doi:10.1039/d6ta04877c)
Supplement: Supplementary file 1 [file TA-014-D6TA04877C-s001.pdf]

Supplementary Information (SI) for Journal of Materials Chemistry A.
This journal is © The Royal Society of Chemistry

Supplementary Information

Visualizing Redox-Driven Metal–Oxide Interface Dynamics in Ni/GDC Electrodes via In-Situ Auger-Meitner Electron Microscopy

Christian Melcher[1], Andreas Nenning[1], Stanislaus Breitwieser[1], Kirsten Rath[1], Luca Balice[2], Jonathan Rollenitz[3], Martin Bram[2], Christoph Rameshan[3], Alexander Karl Opitz[1]

[1]TU Wien, Institute of Chemical Technologies and Analytics
[2]Forschungszentrum Jülich, Institute of Energy Materials and Devices
[3]Montanuniversität Leoben, Chair of Physical Chemistry

1. Materials and Methods

Starting materials

Gadolinium-doped ceria GDC10 (Treibacher Industry AG, lot n. EA35/12.1) and metallic nickel (Toho NF41) are the starting powders selected, based on the previous works of (Haydn et al., 2014) (Rojek-Wöckner et al., 2016) (Bischof et al., 2019). Table 1 reports their particles size distribution (Horiba LA-950 analyzer), before and after sonication in ethanol, and the specific surface area (SSA), measured by nitrogen absorption (Area Meter II, Juwe Laborgeräte GmbH).

Table S 1: particle size distribution and SSA of the GDC10 and Ni powders

	**GDC10 Treibacher**		**Ni Toho NF41**	
	Before sonication	After sonication	Before sonication	After sonication
**D_{10} (µm)**	8	0.05	0.92	0.75
**D_{50} (µm)**	25	0.30	2.59	1.14
**D_{90} (µm)**	50	20.50	44.41	4.24
**S.S.A ($m^2 g^{-1}$)**	7.87		1.43	

GDC10 displays large spherical agglomerates composed by finer primary particles, as shown in Table 1 and in Figure 1 (a-b). Nickel powder has larger spherical primary particles (D_{50} around 1 µm) with a SSA about four times larger than GDC10 (Figure 1 c-d).

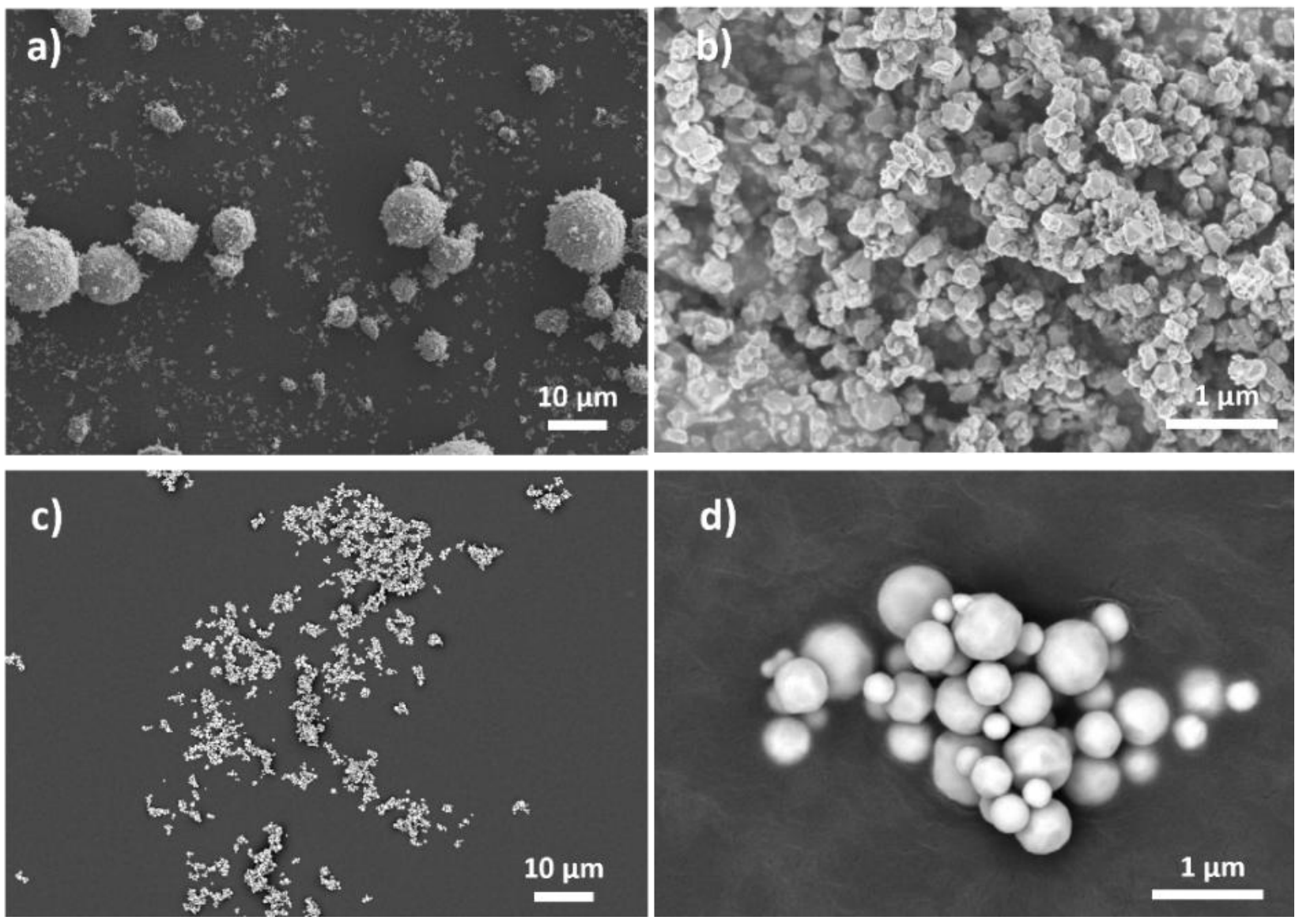

Figure S 1: SEM images of raw powders: GDC10 Treibacher AG (a-b) and Ni Toho NF41 (c-d)SEM Images of Thin-Films

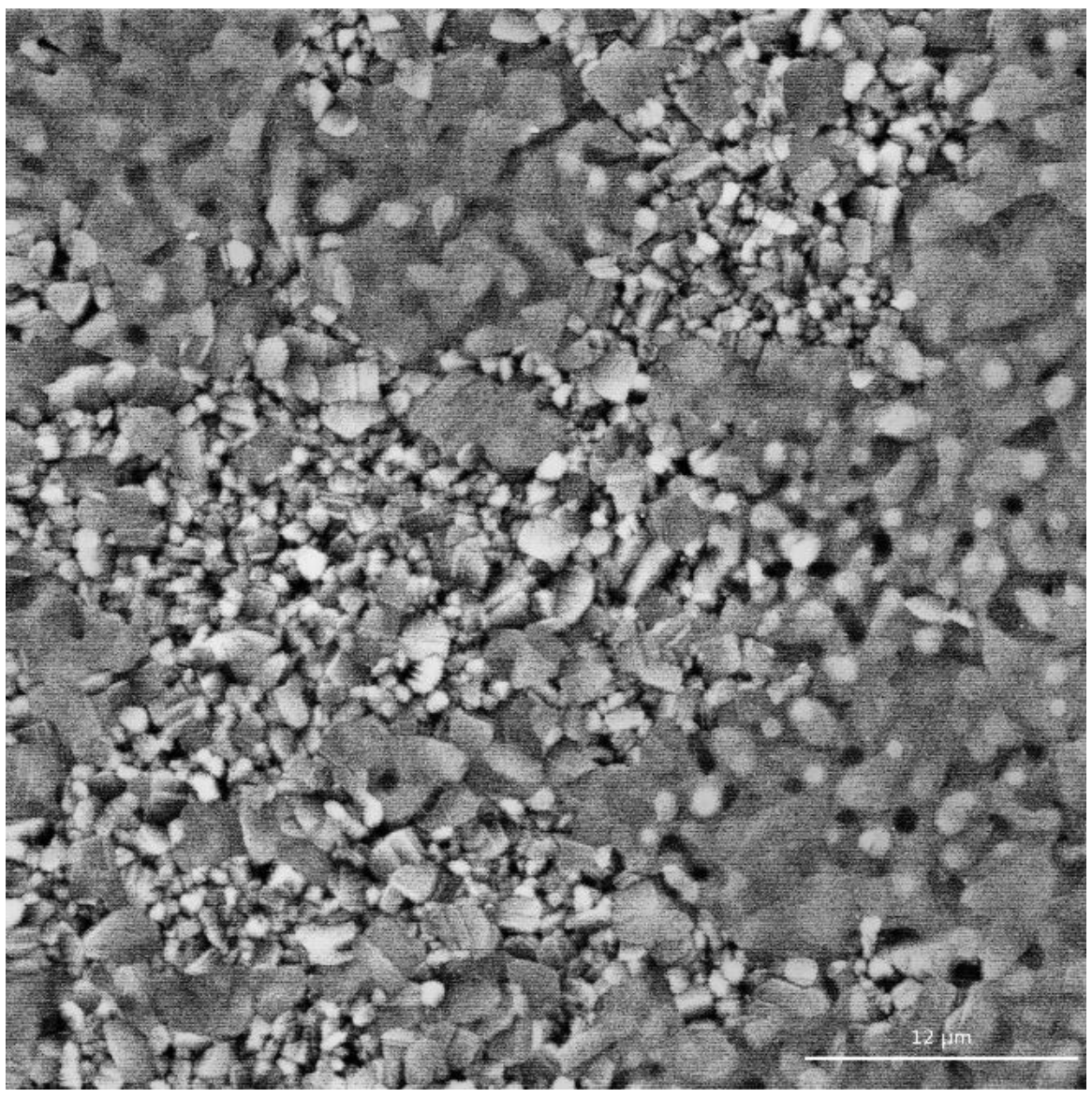

Figure S 2: SEM images of 200 nm Ni layer on GDC at the start of the heatup

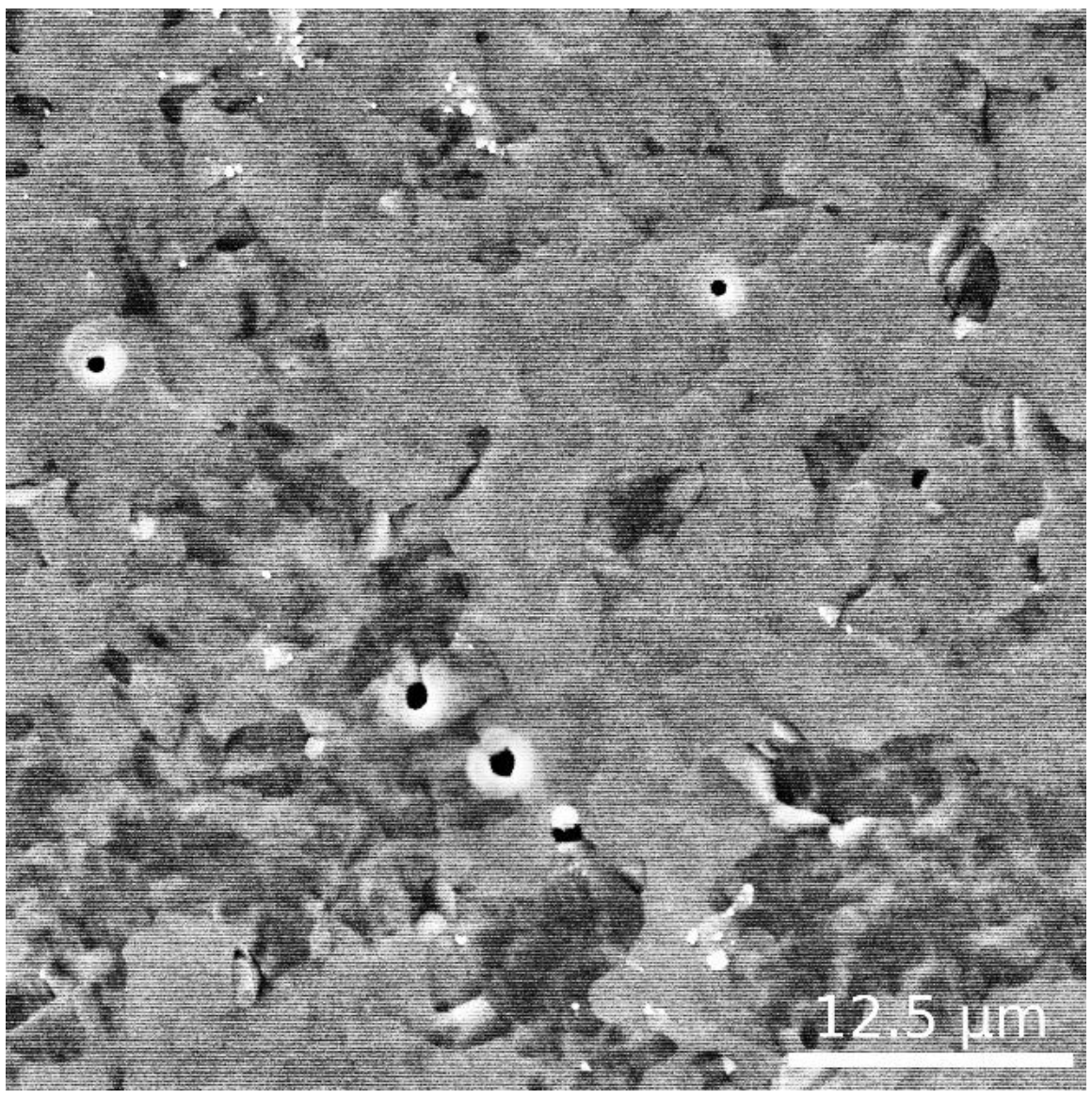

Figure S 3: SEM images of 200 nm Ni layer on GDC after heatup at 750 °C with small pinholes.

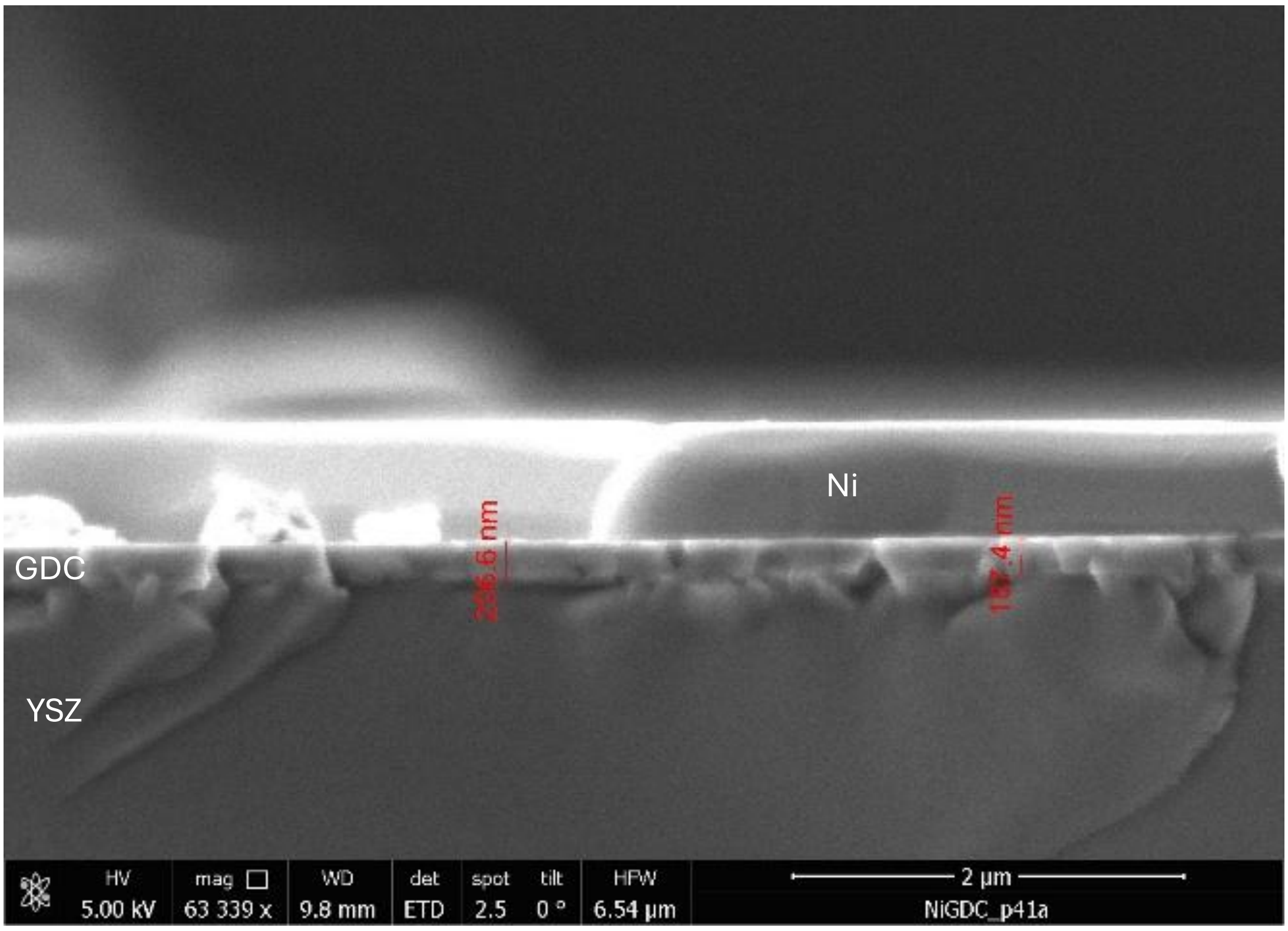

Figure S 4: SEM cross-section of a sample after measurement (type 1).

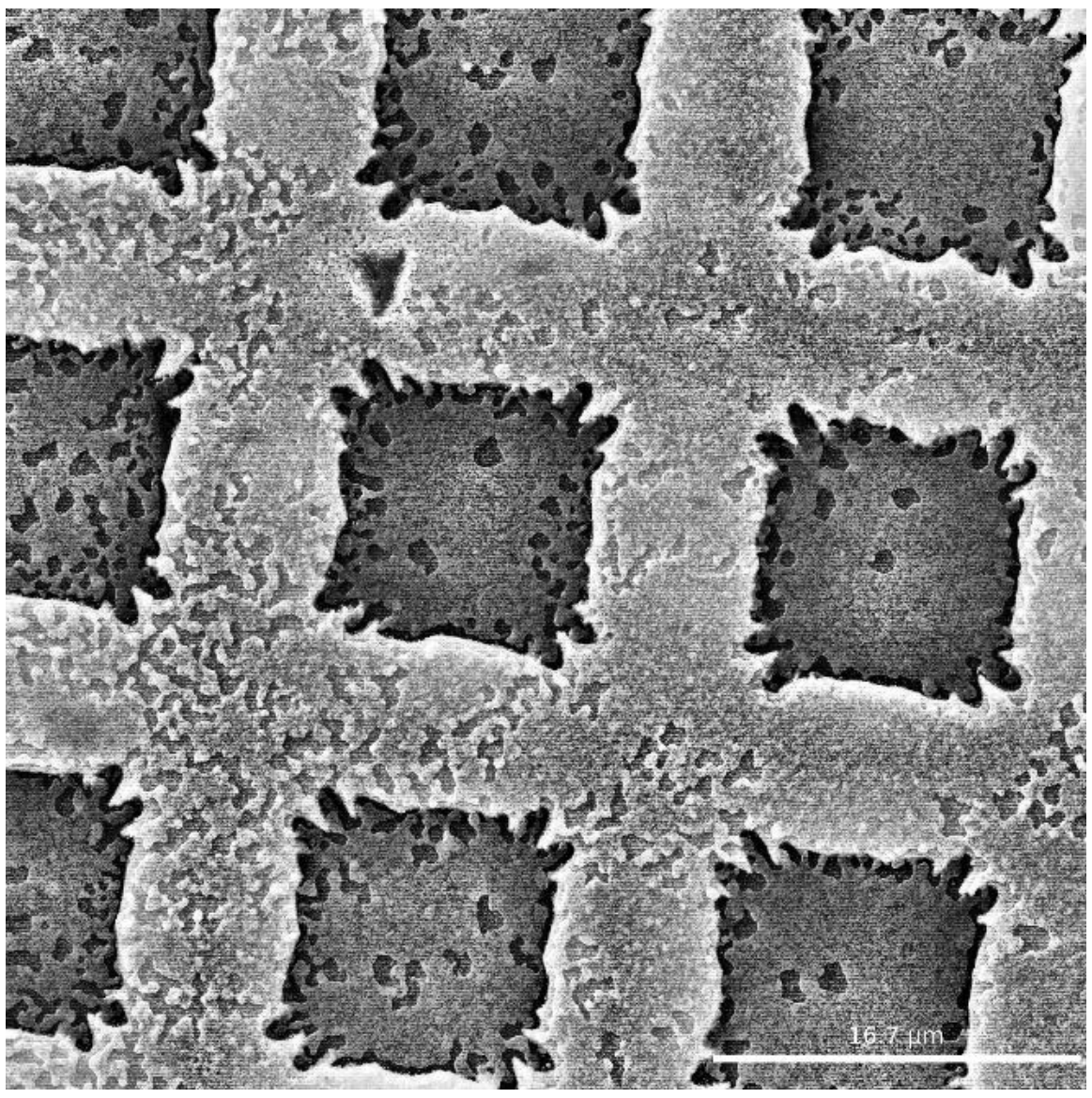

Figure S 5: SEM image of sample type 2 (Ni thickness gradient) in the middle of the sample (higher Ni layer thickness).

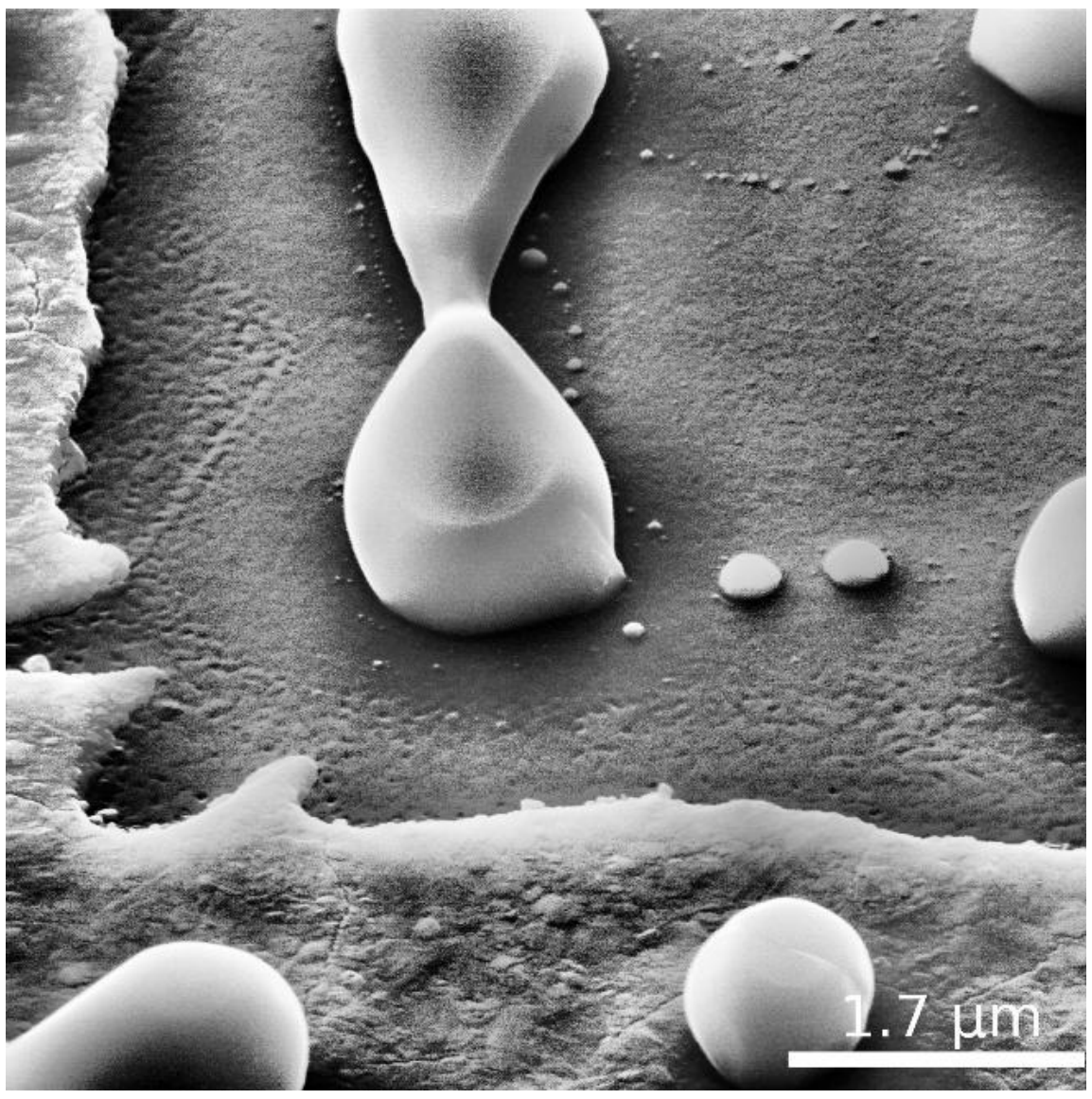

Figure S 6: SEM image of sample type 2 (Ni thickness gradient) closer to the edges of the sample (lower Ni layer thickness).

2. EIS Fittings

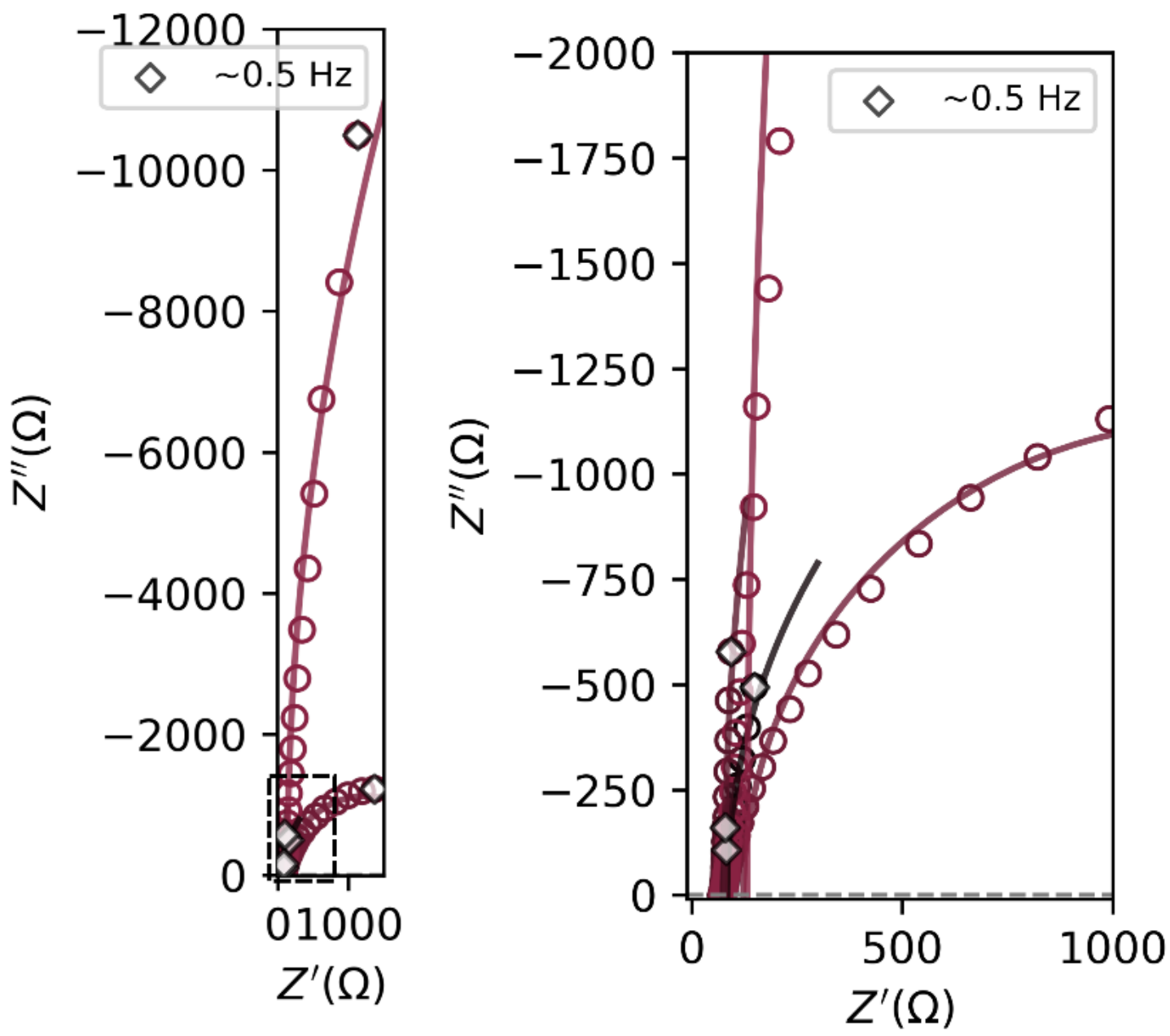

Figure S 7: Exemplary Nyquist-plots of thin-film sample at 750 °C in UHV conditions including R-R||C fitting functions. Left: low magnification, right: zoomed inset.

3. Thin-Film XRD

XRD patterns of Ni/GDC thin films are shown in Figure S 8. The XRD patterns align well with 100-oriented GDC on 100-oriented YSZ with additional peaks from Ni(111) and Ni(200) reflexes. The features marked with a star correspond to spectral line features from the x-ray source.

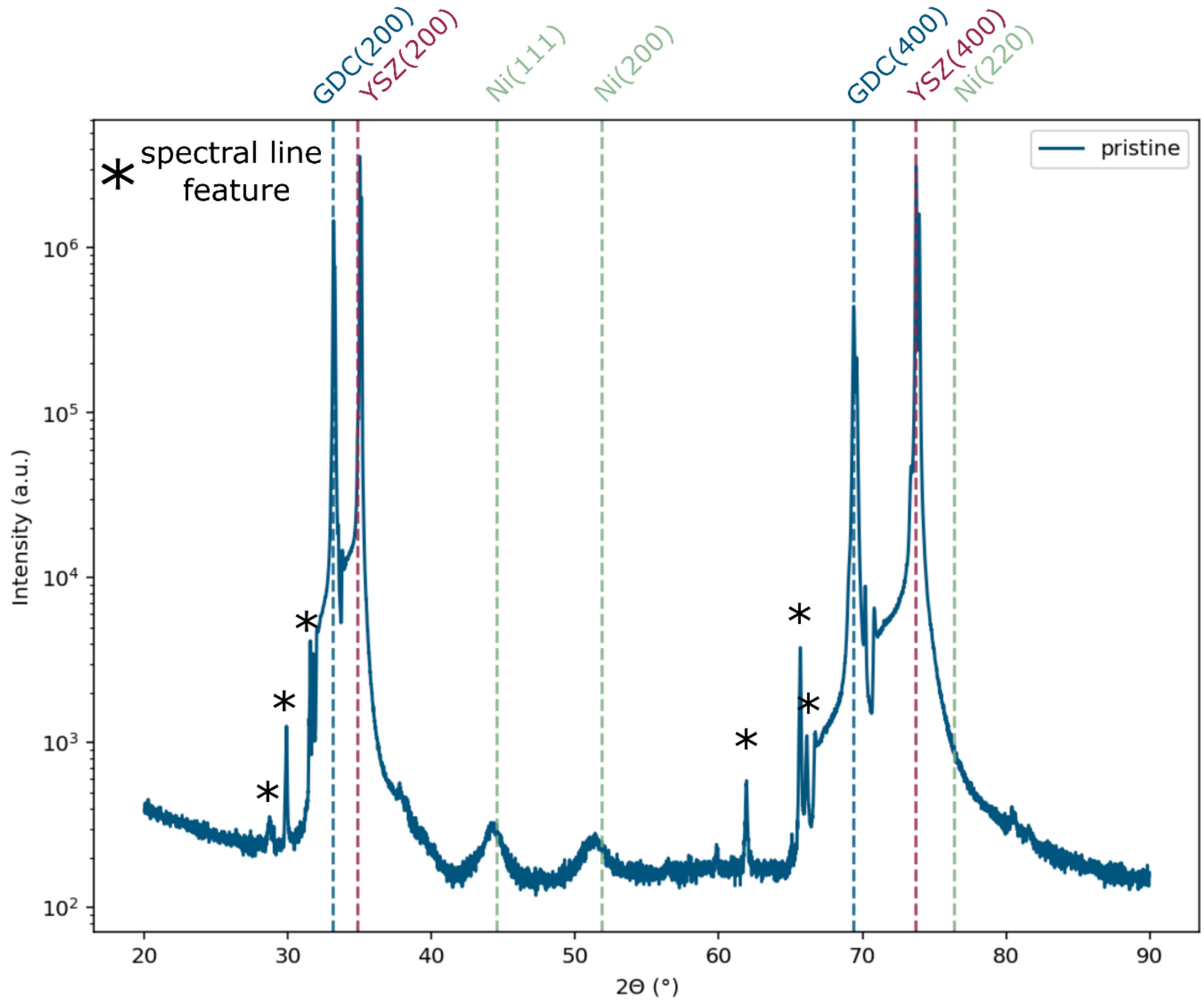

Figure S 8: XRD pattern (Cu Kα, λ = 15.406 nm) of Ni/GDC thin-films on YSZ (100) single crystal right after PLD and magnetron sputtering (pristine): Most intense reflexes are labeled above for GDC (PDF Card - 00-034-0394), YSZ (PDF Card - 00-030-1468) and Ni (PDF Card - 00-004-0850).

4. SEM Segmentation for Quantification

A marker-controlled watershed segmentation was applied to the smoothed SEM images using the local intensity gradient as the flooding topography. Initial ceramic and metal markers were generated from low- and high-intensity percentile thresholds, respectively, followed by morphological cleanup and watershed flooding to separate both phases.

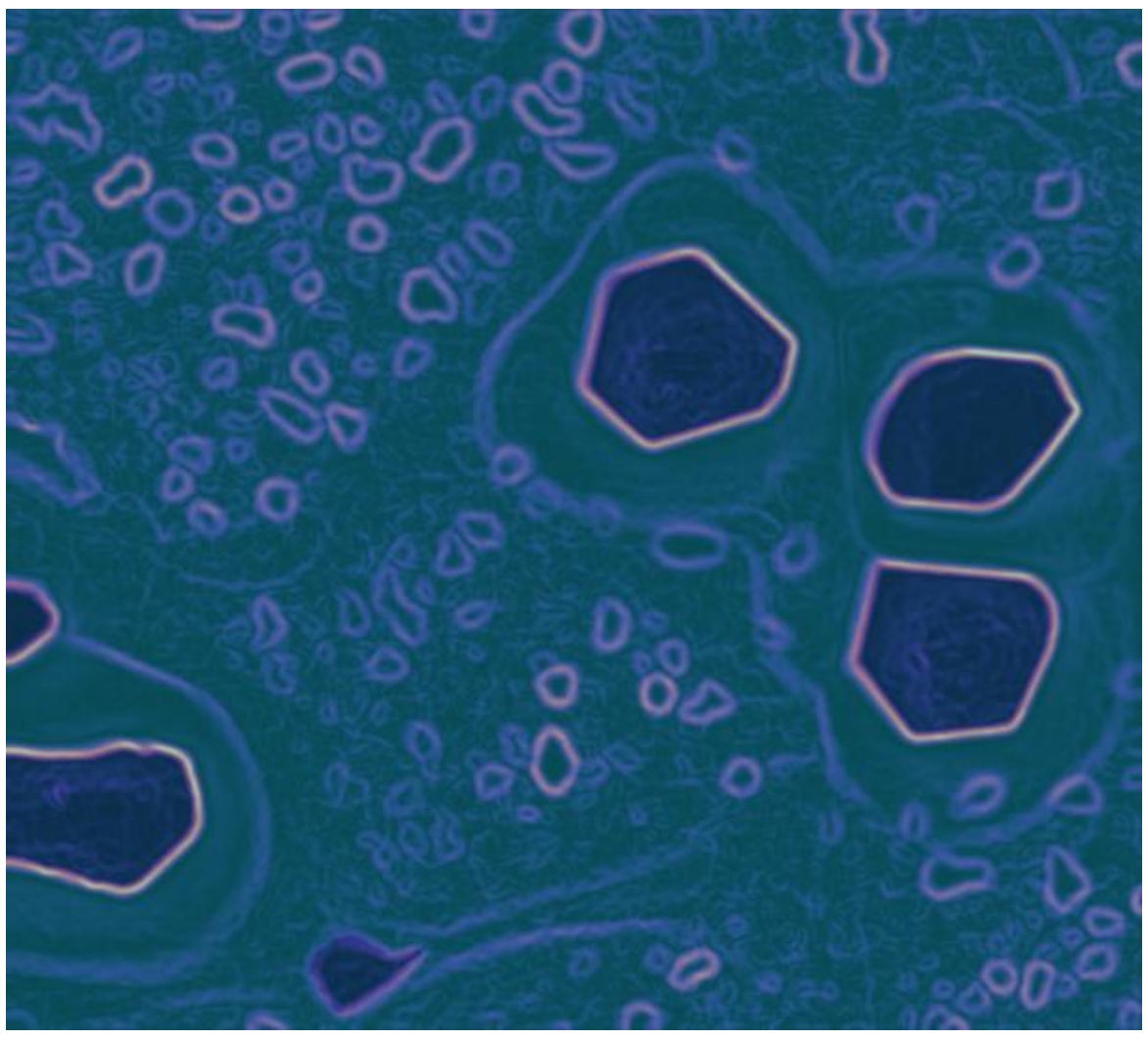

Figure S 9: Local intensity gradient map + segmentation overlay of sample type 1 before redox-cycling.

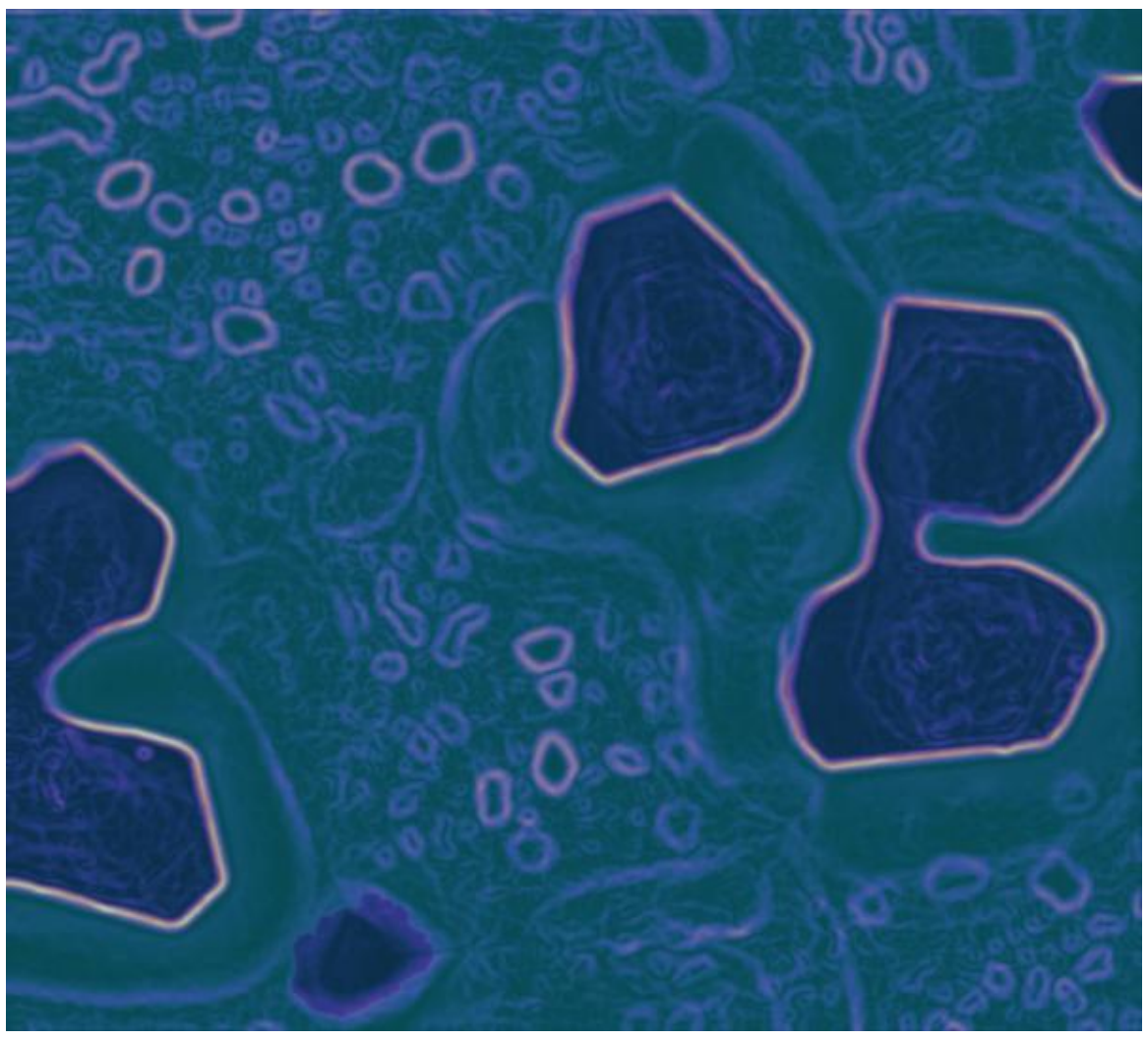

Figure S 10: Local intensity gradient map + segmentation overlay of sample type 1 after redox-cycling.

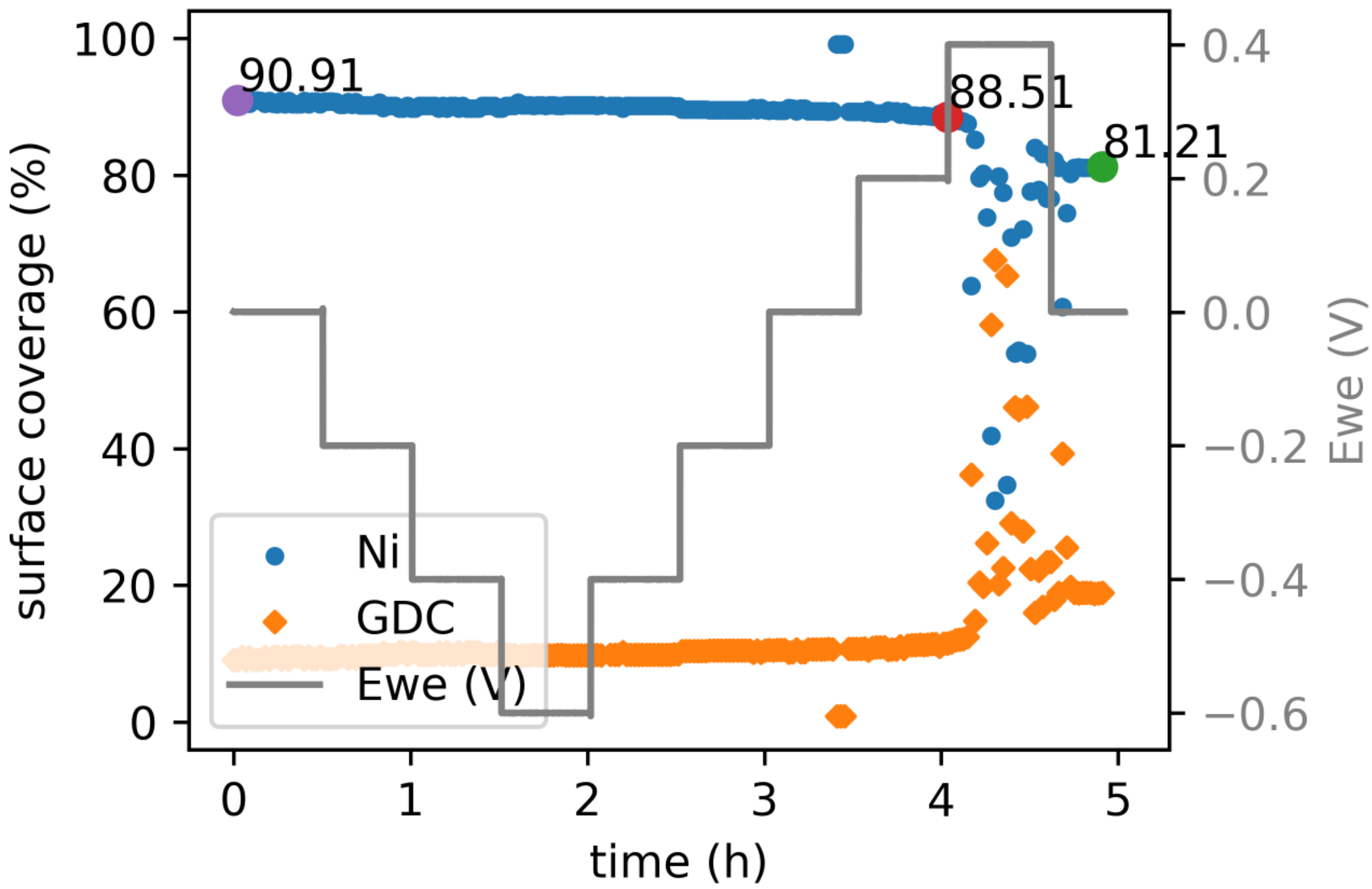

Figure S 11: Resulting surface coverages of Ni and GDC From watershed segmentation. The segmentation fails upon oxidation but recovers a stabile value after the subsequent reduction.

5. EXACT-SEM/AMEM of 3D Porouse Ni/GDC

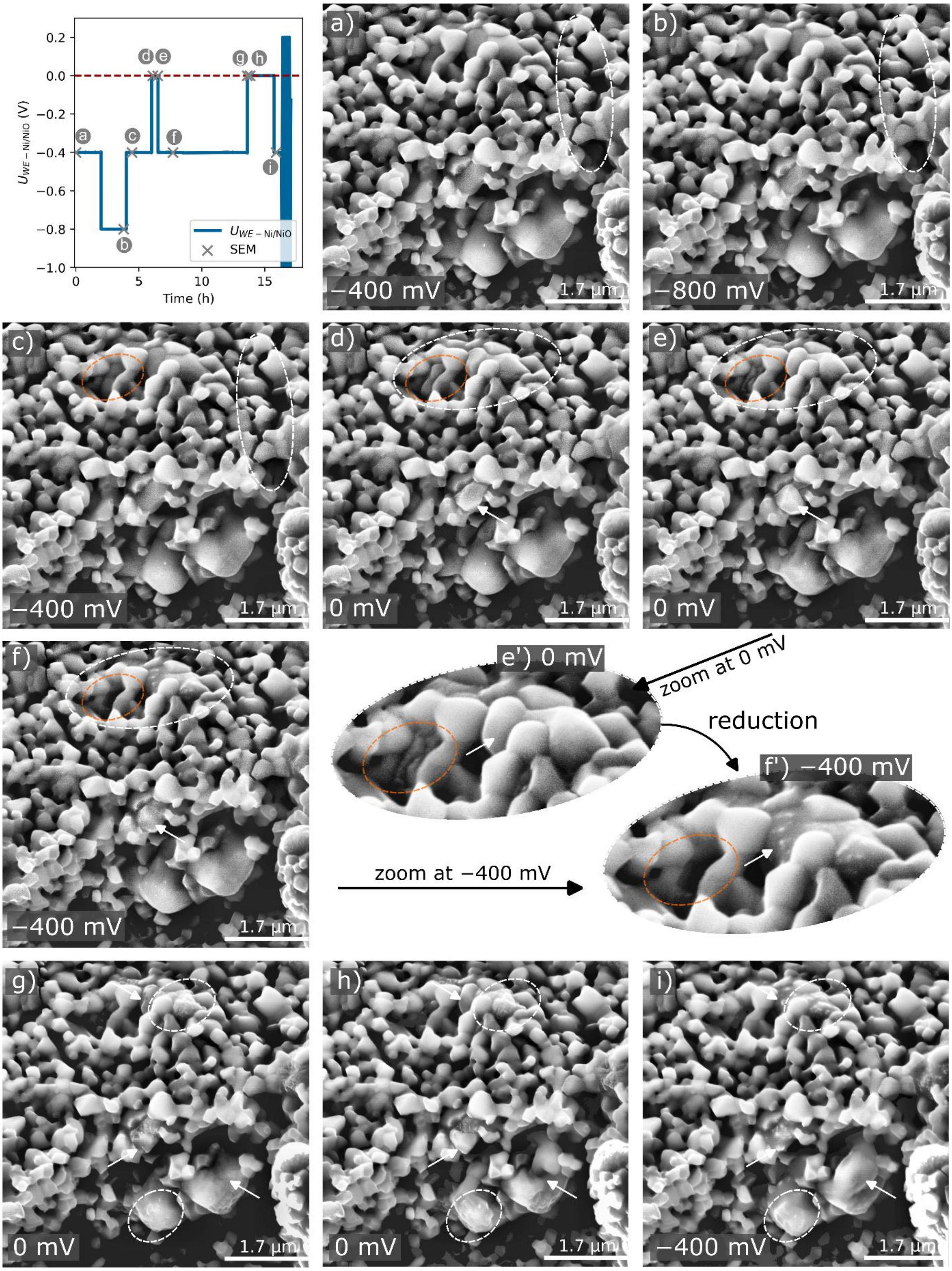

Figure S 12: Voltage program (top left corner) and EXACT SEM images (a – i) of a cleaved cross-section of a porous Ni/GDC electrode at 750 °C upon different voltages UWE – Ni/NiO in UHV. Grey markers in the UWE – Ni/NiO vs. time plot indicating the points for SEM images depicted in a – f. e' and f' depict magnified regions for the first re-reduction from 0 mV to −400 mV vs. Ni/NiO.

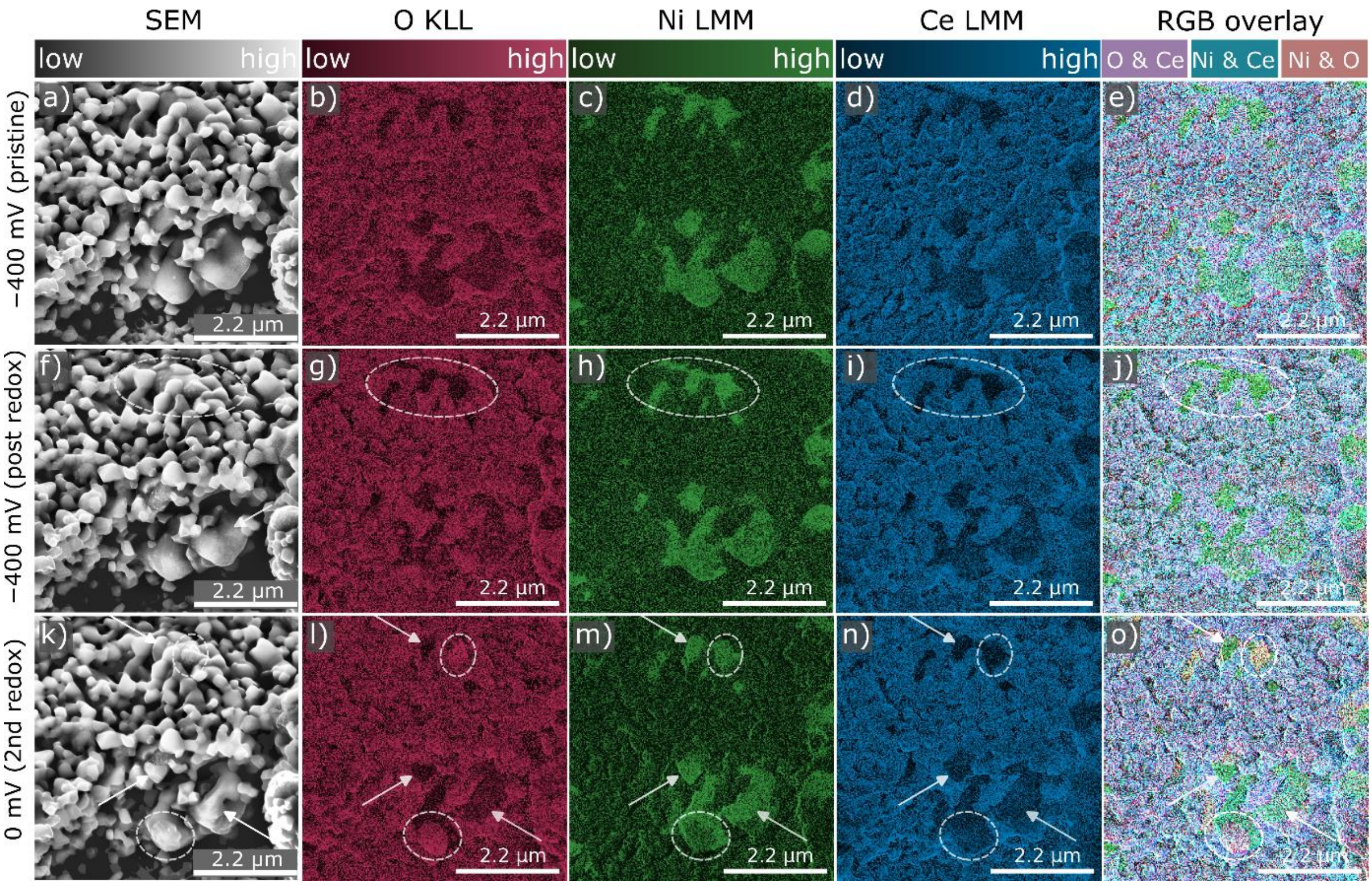

Figure S 13: EXACT-AMEM mappings including SEM images of a cleaved cross-section of a porous Ni/GDC electrode at 750 °C upon different voltages U WE – Ni/NiO in UHV of: the pristine sample at −400 mV after heating up (a – f), the sample after the first redox-cycling at −400 mV (f – j), and the sample upon the second oxidation (k – o).

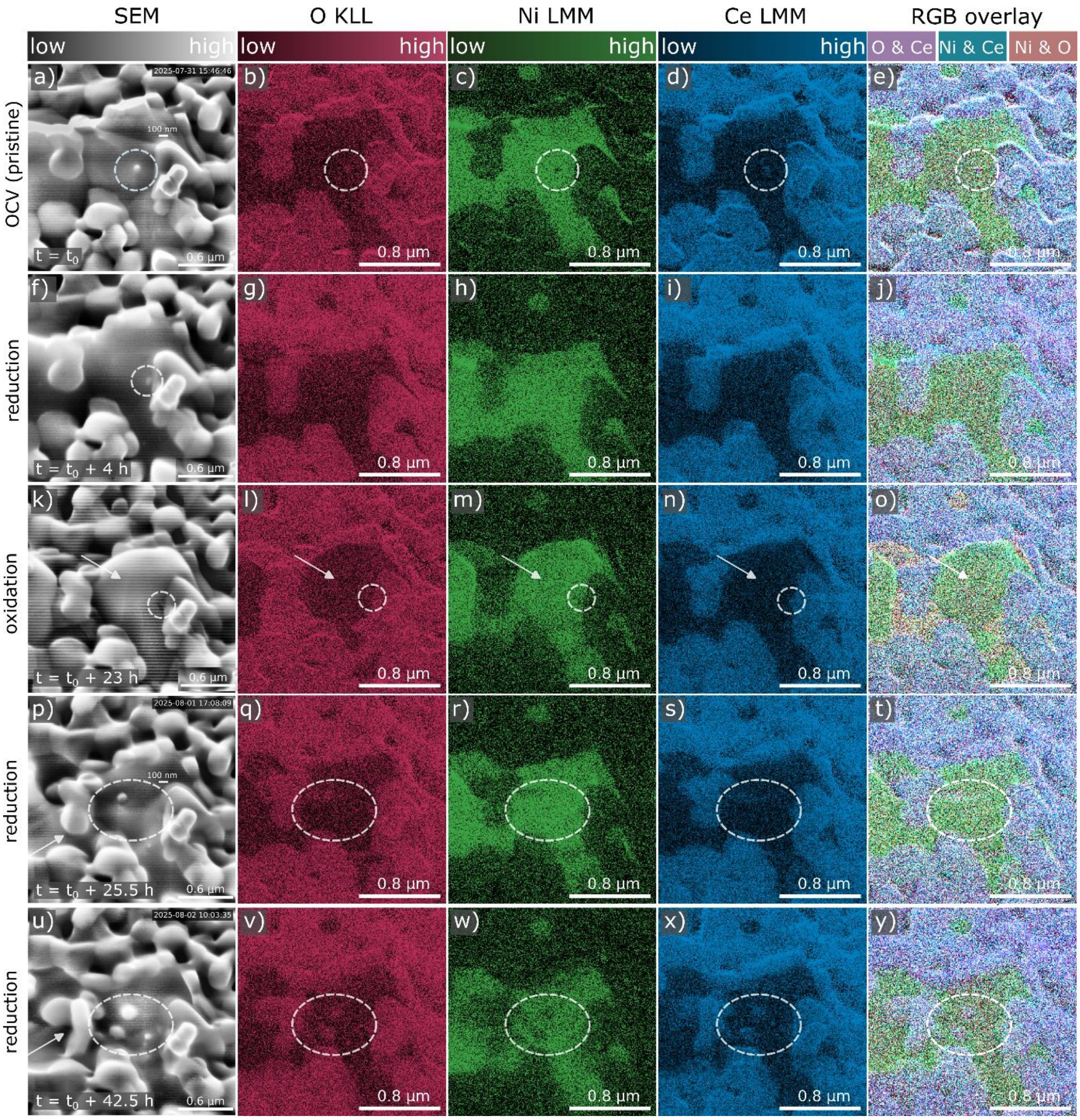

Figure S 14: EXACT-AMEM mappings including SEM images of a second cleaved cross-section of a porous Ni/GDC electrode at 750 °C upon different conditions in UHV: the pristine sample at OCV after heating up (a – f), the sample under reducing conditions (f – j), under oxidizing conditions (k – o), and the sample upon the re-reduction(p – y).
